# Utilising causal inference methods to estimate effects and strategise interventions in observational health data

**DOI:** 10.1371/journal.pone.0314761

**Published:** 2024-12-30

**Authors:** Bao Duong, Manisha Senadeera, Toan Nguyen, Melanie Nichols, Kathryn Backholer, Steven Allender, Thin Nguyen

**Affiliations:** 1 Applied Artificial Intelligence Institute (A2I2), Deakin University, Geelong, Australia; 2 Global Centre for Preventive Health and Nutrition (GLOBE), Faculty of Health, Deakin University, Geelong, Australia; University of South Australia, AUSTRALIA

## Abstract

Randomised controlled trials (RCTs) are the gold standard for evaluating *health interventions* but often face ethical and practical challenges. When RCTs are not feasible, large observational data sets emerge as a pivotal resource, though these data sets may be subject to bias and unmeasured confounding. Traditional statistical (or non-causal) learning methods, while useful, face limitations in fully uncovering causal effects, i.e., determining if an intervention truly has a direct impact on the outcome. This gap is bridged by the latest advancements in causal inference methods, building upon machine learning-based approaches to investigate not only population-level effects but also the *heterogeneous* effects of interventions across population subgroups. We demonstrate a causality approach that utilises *causal trees* *and forests*, enhanced by weighting mechanisms to adjust for confounding covariates. This method does more than just predict the overall effect of an intervention on the whole population; it also gives a clear picture of how it works differently in various subgroups. Finally, this method excels in strategising and optimising interventions, by suggesting precise and explainable approaches to targeting the intervention, to maximise overall population health outcomes. These capabilities are crucial for health researchers, offering new insights into existing data and assisting in the decision-making process for future interventions. Using observational data from the 2017-18 Australian National Health Survey, our study demonstrates the power of causal trees in estimating the impact of exercise on BMI levels, understanding how this impact varies across subgroups, and assessing the effectiveness of various intervention targeting strategies for enhanced health benefits.

## 1 Introduction

Randomised controlled trials (RCTs) are widely considered the gold standard for evaluating the impact of interventions. In these trials, participants are randomly assigned to groups, such as treatment or control [1]. Throughout this paper, “treatment” is used to mean an intervention or exposure that is expected to have a causal impact on the outcome of interest. In various fields of health research, such “treatments” may include pharmaceutical, surgical, behavioural, environmental or other interventions [2, 3]. With a sufficiently large sample size, random allocation in RCTs should balance participant characteristics across the groups, minimising the risk of bias due to unmeasured confounding. This balance allows any outcome differences to be confidently attributed to the treatment itself [4, 5]. However, RCTs are not always be feasible due to factors such as cost, physical constraints, or ethical issues [5, 6].

In the absence of RCTs, researchers may resort to *non-randomised* intervention designs, including quasi-experimental studies and natural experiments. In such cases, traditional statistical (non-causal) approaches conduct statistical tests based on the differences in the outcome between the treatment (intervention) group and the control group [7, 8]. These methods, while practical, when used with non-randomised data, may be inherently more prone to significant (unidentified) biases due to the potential for *unequal* distribution of population characteristics between study groups [9, 10]. These biases stem from *unadjusted covariates* or *confounders*—factors that affect both the choice of treatment and the outcome, creating spurious dependencies between them [11, 12]. For example, in a study evaluating a health-related treatment that is not randomly assigned, individuals who are more health-conscious or already experiencing health issues might be more inclined to choose the treatment. Conversely, those less concerned about their health may opt out. This self-selection introduces biases that can skew the results and obscure the true effect of the treatment.

Causal inference techniques provide a means to evaluate treatment effects by effectively controlling for confounding factors, thereby removing associated biases [5, 10]. We examine the Average Treatment Effect (ATE), which assesses the overall impact of a treatment at the population level [9]. ATE represents the expected change in outcomes from applying the treatment across the entire population, compared to the scenario where the control condition is universally implemented. ATE, however, measures overall impact in a population, and does not explain how different subgroups respond uniquely to an intervention. The presence of *heterogeneous* treatment effects is common, influenced by diverse individual characteristics across subgroups of a target population [13, 14]. While an intervention might be effective and beneficial for some subgroups, it could have no effect or even be detrimental in others. Therefore, assessing average treatment effects at the subgroup level, known as Conditional Average Treatment Effects (CATE)—for example, analysing the intervention’s impact based on age, gender, or socio-economic status—enables more strategic and precise recommendations [14, 15]. This is due to a better understanding of subgroup responses [16]. By identifying these varied treatment effects, we can develop more effective treatment strategies to enhance resource allocation efficiency and overall benefits.

In this paper, we demonstrate a framework for causal inference methods, enhanced by machine learning techniques, which i) can control for confounding factors to estimate average treatment effects in both randomised and non-randomised (observational) health studies; ii) utilise causal tree and forest methods to autonomously identify heterogeneous treatment effects within population subgroups, offering interpretable insights; and iii) evaluates potential intervention targeting strategies, identifying the most beneficial sub-population targeting approach to maximise treatment benefits across the entire population. While current causal methods are applied to observational health data [17–21], our use of causal trees stands out for its explainability and proficiency in estimating heterogeneous treatment effects. This approach not only enhances our understanding but also assists health researchers in formulating strategic, optimal treatment policies by effectively identifying varied treatment effects at both the population and subgroup levels.

We utilise a subset of cross-sectional data from the 2017–18 Australian National Health Survey (NHS) [22]. Focusing on adults (aged 18+), we demonstrate how causal inference methods can be applied to estimate the effects of an exposure (*whether one does enough exercise or not*) on an outcome (*body mass index (BMI)*) from this observational dataset. The exposure (intervention / treatment) is not randomised but self-selected by the individual and the distribution of covariates is not expected to be even between the ‘intervention’ and ‘control’ groups. The NHS data used in the demonstration are cross-sectional, meaning that the temporal sequence of variables is not guaranteed, and therefore reverse causality cannot be excluded (i.e., a high or low BMI may be the cause of exercise levels) or adjusted confounding factors might have occurred after the exposure or the outcome. However, this should not be a concern in longitudinal or experimental data where there is a temporal separation between the observation of exposure and outcome.

This study illustrates the application of causal inference machine learning methods to address several key questions in observational health data:

1. What is the average treatment effect at the population level? (Section 4.2)
2. Is there heterogeneity in treatment effect across subgroups? (Section 4.3.1)—for which we employ causal tree and forest methods.
  (a) Are there differences in treatment effects between pre-specified groups? (Section 4.3.2)
  (b) Can we discover subgroups automatically? (Section 4.3.3)
3. Upon identifying heterogeneity, how could strategies, supported by the interpretability of causal trees, targeting the treatment to population sub-groups be used to optimise the intervention effect across the entire population? (Section 4.4)

## 2 Background

### 2.1 Current methods for treatment effect estimation

Even though RCTs are the gold standard for measuring the effect of interventions, they are typically expensive and difficult to execute, especially when attempting to include sufficient participants and power to analyse different effects given variations in individual, population or intervention characteristics. Additionally, power calculations for these clinical trials typically only focus on a limited number of population sub-groups, and sometimes, certain groups (like women or specific age groups) might not be included in the trial design at all.

In epidemiology and biostatistics, to understand how treatment effects differ across pre-defined sub-groups in a RCT a standard approach, known as “effect modification”, is to add a potential modifying factor, typically just one, along with its interaction with the treatment, to the statistical model used for estimating the overall treatment effect. However, this method becomes impractical when dealing with a substantial number of factors that define the sub-groups (such as age, gender, health status) or when there are many levels within these factors, due to the “curse of dimensionality” [23]. Essentially, as the number of factors increases, the number of required observations to accurately estimate effects in each unique combination of sub-group factors grows exponentially.

Due to practical sample size limitations, reliably assessing treatment effect heterogeneity (via effect modification) using standard methods is only feasible for a small number of subgroups. This constraint limits our capacity to identify specific combinations of participant characteristics (sub-groups) that significantly impact the treatment effect. Consequently, this limitation affects our capability to: i) accurately target subpopulations most likely to gain substantial benefits from the intervention; ii) prevent resource waste by not targeting sub-groups that are unlikely to see significant benefits; and iii) avoid applying interventions in sub-populations where such interventions could potentially be harmful.

In the era of big data, with a vast amount of mostly observational health data, it becomes challenging for health data users to manually identify complex patterns for informed decision-making [21, 24]. As a result, the significance of Machine Learning (ML) methods in this context is clearer than ever. Especially in healthcare, ML algorithms have proven to be highly effective in solving a range of problems, such as, e.g., heart disease detection [25, 26], diabetic disorder analysis [27–30], cancer identification [31–33], and thyroid diagnosis [34, 35].

### 2.2 Causal inference

Despite the success of ML, conventional ML methods merely focus on finding the statistical associations in data, which does not imply causal relationships [5, 9, 10]. Therefore, causal inference methods have been developed as an extension to ML to account for the causal queries [5, 35–37]. Existing causal methods have been applied to analyse observational health data [17–21]. However, our use of causal trees stands out for its ability to explain and estimate treatment effects effectively. This, in turn, provides valuable support for healthcare practitioners in devising treatment strategies.

The causal effect of a treatment on a single individual *i* at a given time point is the difference between two potential outcomes, the potential outcome if the individual receives treatment *Y*_*i*_(*W*_*i*_ = 1) and the potential outcome if they do not receive treatment *Y*_*i*_(*W*_*i*_ = 0); where *Y* indicates the outcome and *W* the treatment [38]. The main challenge of causal inference is that both potential outcomes cannot be simultaneously observed because multiple treatments assignment cannot take place at the same time for the same individual. While estimating treatment effects at an individual level is not feasible, it is possible to estimate causal effects of interest *at the population or subgroup level*, as long as certain below assumptions are met [10, 39]. We give a formal definition of causal effects at these levels in Section 3.

For an individual labeled *i*, let *W*_*i*_ represent the treatment actually received, *Y*_*i*_ their actual observed outcome, and *Y*_*i*_(0) and *Y*_*i*_(1) the potential outcomes that have been occurred if, hypothetically, the individual had received treatment *W* = 0 or *W* = 1, respectively. Firstly, the *consistency* assumption states that an individual’s potential outcome under the observed treatment is equal to the observed outcome, and can be formulated as:

$$\begin{array}{c} Y_{i}\equiv Y_{i}\left(W_{i}\right)=\{\begin{array}{cc} Y_{i}\left(0\right) & \text{if}\;W_{i}=0\\ Y_{i}\left(1\right) & \text{if}\;W_{i}=1 \end{array} \end{array}$$ (1)

$$\begin{array}{c} =W_{i}\cdot Y_{i}\left(1\right)+\left(1-W_{i}\right)\cdot Y_{i}\left(0\right) \end{array}$$ (2)

The second key assumption for causal inference is referred as to *ignorability*, *exchangeability* or no-unmeasured confounders. It states that potential outcomes are conditionally independent of the treatment, given a set of measured covariates. If *X* denotes the set of confounders, the assumption is formulated as (*Y*(1), *Y*(0)) ╨ *W* ∣ *X*. This assumption means that all possible factors that are common causes of both the treatment choice and the outcome (confounders) are available in the dataset.

The assumptions of ignorability and consistency are fundamental and widely accepted in the field of causal inference [1, 5, 9, 20, 40], playing a crucrial role in enabling the estimation of treatment effects from observational data. In our study, while these assumptions are inherently untestable, we ensure their plausibility through careful study design and methodology. The third assumption, concerning *positivity* or *overlap*, is rigorously tested using our experimental health data, further solidifying the reliability and applicability of our causal inference methods in practical research scenarios.

The key assumption of *overlap* or *positivity* states that the conditional probability of receiving a specific treatment (propensity score) should always be greater than 0 and less than 1 for each subgroup defined by various combinations of covariates [41]. This ensures that within every set of covariates, there are individuals who have received both the treatment and control, allowing for effective comparison across all covariate combinations. For a validation of this assumption in our study, please refer to Section 4.

### 2.3 Causal trees and forests

The causal tree and causal forest frameworks [15, 42] offer a wide range of machine learning tools that are suited to address causal inference questions and suitable for both randomised and non-randomised data. These methods apply recursive partitioning to allow the discovery and estimation of heterogeneous treatment effects in both RCT and observational data.

**Fig 1:**
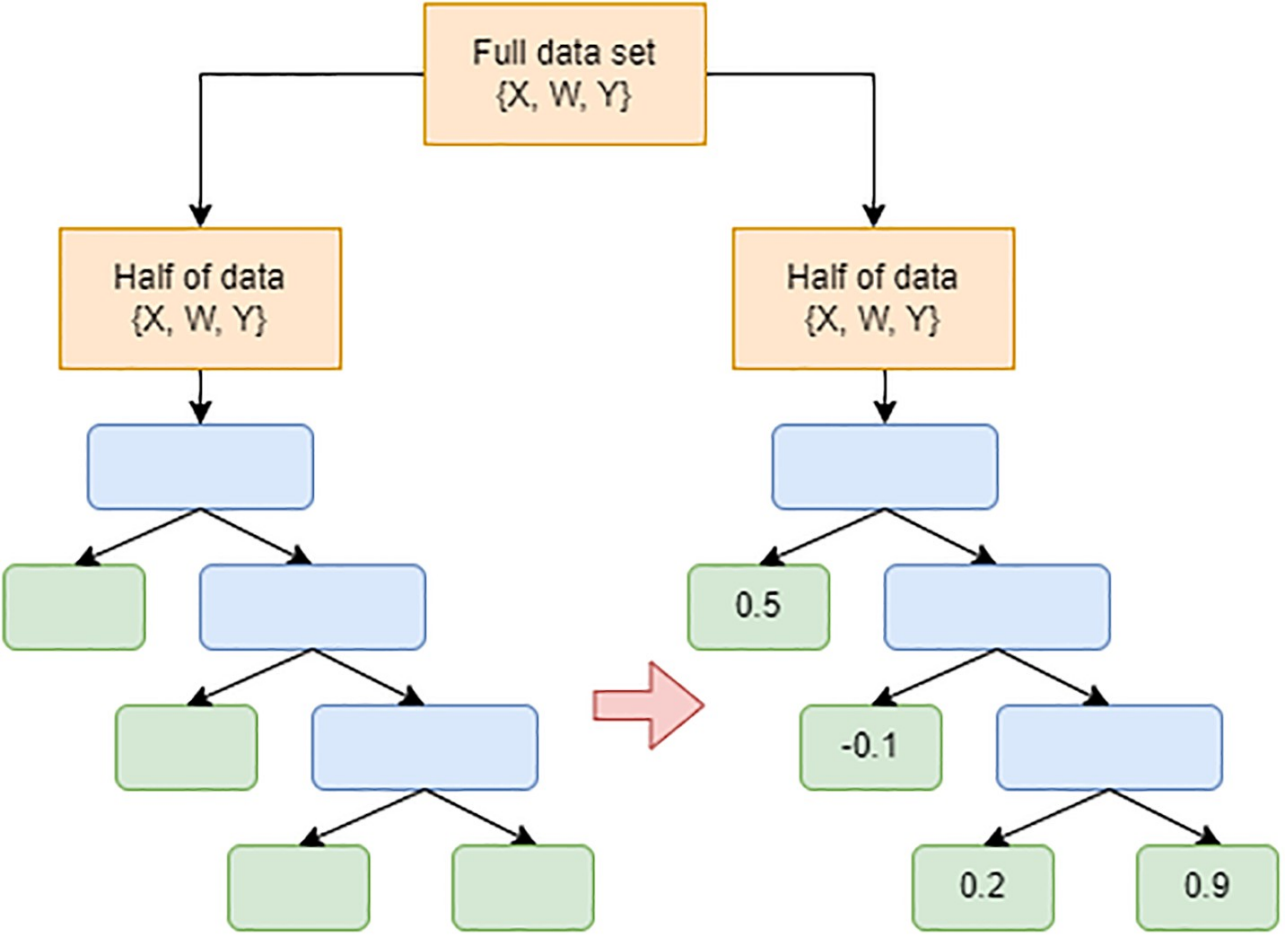
To build a causal tree, the full data set is randomly split into two halves. The first half is used to construct the tree architecture and splitting rules, while the second half is used to estimate the treatment effect at each leaf (subgroup).

Athey and Imbens innovated the use of causal trees, an extension of regression tree methods, to estimate heterogeneous treatment effect [42]. In their approach, known as “honesty trees”, a dataset is divided into two parts: one for constructing the tree’s structure and the other for estimating treatment effects in the subgroups identified by the leaves of the trees (see Fig 1). To create the structure of the tree, a branch splitting criterion is applied that seeks to maximise the heterogeneity in treatment effect between groups, using the first subset of the data. The second subset is then used to produce treatment effect estimates in the generated subgroups, thereby preventing overfitting and removing bias in the estimates. The honesty tree approach prevents overfitting and allows heterogeneous treatment effects to be found without specifying the subgroups beforehand.

A key distinction between causal trees and traditional decision trees is their outputs: while decision trees merely predict *conditional* *expected outcomes* (like anticipated BMI based on health information), causal trees calculate *conditional average treatment effects*, namely, the *expected change* in BMI if all individuals in a subgroup were treated versus untreated.

Causal trees effectively segment the population using covariates, creating subgroups through clear and interpretable rules, without compromising predictive accuracy. This process serves as a practical guide for practitioners in targeting treatments. However, a challenge with causal trees lies in the variability of the results, as different random data splits can lead to the generation of varying honesty trees, thus affecting the reproducibility of the subgroup sets.

**Fig 2:**
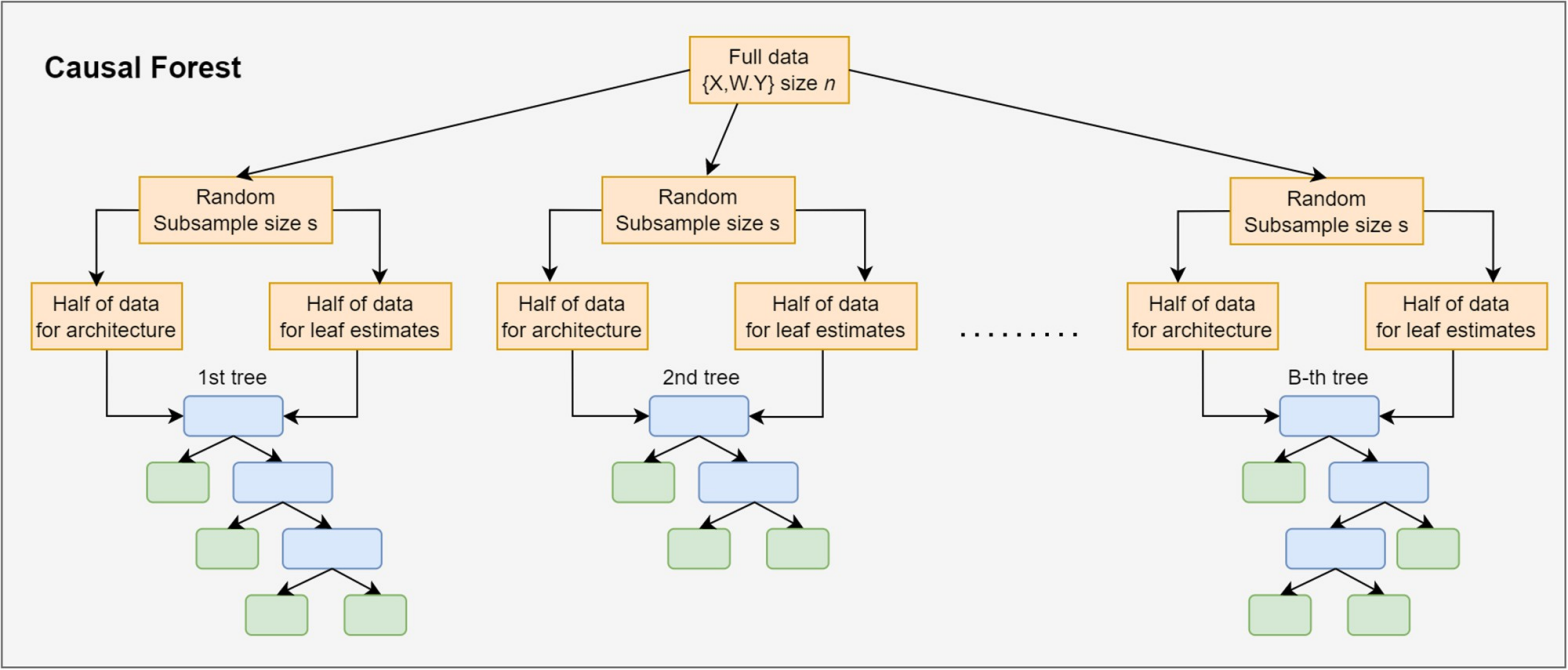
To build a causal forest from the full data (size *n*), *B* subsamples of size *s* are randomly selected. These subsamples are used to build *B* honest causal trees, as described in Fig 1. The estimated treatment effect for a specific individual is calculated as the average of that individual’s predictions across all *B* trees in the model. For out-of-the-bag predictions, the estimation only utilises trees that did not have the test individual in their training data set.

To overcome these limitations, the concept of a causal forest has been introduced [15]. Essentially, a causal forest consists of numerous causal trees, each analysing the data from a unique “view”—using different subsets of observations and covariates. The overall prediction of the conditional average treatment effects (CATE) for an individual in a causal forest is derived by averaging out the predictions from each of these individual causal trees. Fig 2 illustrates the collection of causal trees within a causal forest. To estimate an individual’s treatment effect, the model averages predictions from trees that did not include that individual’s data during training, thereby further reducing bias.

Causal forests offer several advantages. They create smoother decision boundaries, leading to more robust estimations, and allow for confidence intervals based on covariates. These forests also enable predictions of personalised treatment effects. Unlike a single tree, they avoid issues with small sample sizes by re-randomising the subsets for each tree in the forest. Additionally, causal forests maintain the interpretability of causal trees, serving as a practical tool for practitioners in customising treatment strategies. They also provide insights into the role of each covariate in creating treatment effect heterogeneity, determined by tracking the frequency of each covariate’s involvement in branch splitting across the trees.

### 2.4 Related works

#### 2.4.1 Application of causal trees and forests to health data

The goal of discovering subgroups with varying treatment effects has resulted in a number of studies [43–45], yet these methods do not focus on hypothesis testing in their design. Causal tree and causal forest methods have been applied to a number of problems ranging from discovering risk of recidivism under varying degrees of police supervision [46], the effect of time-of-use pricing on household electricity consumption [47], improving student achievement through nudge interventions [48], and the effectiveness of summer job programs in disadvantaged youth [49]. In the context of observational health related data, causal forest techniques have been applied to estimate the impact of residing in different regions of Europe on the prevalence of diabetes [50], and the impact of health insurance policies on infant mortality in Indonesia [51], as well as children’s health status in rural China [52]. Our study utilises the interpretability and strong estimation abilities of causal trees and forests to predict heterogeneous causal effects within data subgroups. This not only aids health practitioners in devising strategic and optimal policies (based on the estimated effects) for diabetes prevention but also contributes to maintaining a healthy BMI index among the population.

#### 2.4.2 Causal inference

Various causal inference approaches can be utilised to estimate heterogeneous causal effects [5, 53, 54]. Linear or LASSO regression [55, 56] offers strong interpretability through its coefficients but struggles with complex heterogeneity due to its linear assumptions and reliance on manually specifying interactions between variables. In contrast, our causal trees and forests also provide interpretability by tracing decision paths for treatment effects while handling non-linear relationships without relying on predefined functional forms [15, 42, 57]. Propensity score [58, 59], or backdoor adjustment methods [5] control confounding bias by balancing treated and control groups, but they are not well designed for handling complex heterogeneity. In our framework, integrating propensity scores with causal trees and forests effectively addresses both confounding bias and heterogeneity. Front-door adjustment [60, 61] though robust to hidden confounders, relies on the presence of mediator variables between the treatment and outcome, which may be unavailable in some observational health datasets. Instrumental variables [62, 63], while also robust to hidden confounders, estimate effects for a narrow group affected by the instrument, limiting their generalisability. In contrast, causal trees provide broader treatment effect estimates across subgroups, making them more adaptable to heterogeneous data. Bayesian causal inference methods [64, 65] offer a probabilistic framework that estimates the distribution of heterogeneous treatment effects, providing uncertainty with each prediction rather than just point estimates like other methods. However, they can be computationally demanding and challenging to explain to non-expert audiences in health research.

In sum, our tree-based causal inference framework, combined with propensity score methods, effectively balances handling complex heterogeneous treatment effects and interpretability, making it ideal for providing actionable insights to healthcare practitioners from observational health data.

## 3 Method

**Notations**. Let $D=\{(X_{i},W_{i},Y_{i}){\}}_{i=1}^{n}$ be the data set of *n* individuals, where each individual is indexed by *i* = 1, …, *n*. $X_{i}\in R^{d}$ is the covariate vector of *d* attributes. The treatment variable is indicated by *W*_*i*_ which we assume to be binary, i.e., *W*_*i*_ ∈ {0, 1} where *W*_*i*_ = 1 means that individual *i* belongs to the treated group, and *W*_*i*_ = 0 means the individual is in the control group. Lastly, *Y*_*i*_ is a scalar variable indicating the observed outcome for individual *i*.

In our study, the dataset consists of individual patients, with each patient’s attributes such as work hours, education level, and food consumption serving as components of the covariate vector. The exercise level is considered the treatment variable; patients engaging in moderate to high levels of exercise are marked as receiving treatment (exercise = 1), while those with no or minimal exercise are categorised as not receiving treatment (exercise = 0). Our primary focus is to evaluate the causal effects of exercise on BMI, exploring whether regular physical activity can reduce BMI levels and help in preventing obesity, taking into account the potential impact of various patient characteristics on BMI beyond the exercise treatment.

### 3.1 Average Treatment Effect (ATE)

The average treatment effect (ATE) is defined in terms of potential outcomes as:

$$\begin{array}{c} \tau =E[Y_{i}(1)-Y_{i}(0)], \end{array}$$ (3)

where *Y*_*i*_(1) is the potential outcome of the *i*-th individual has they been allocated into the treatment group, and *Y*_*i*_(0) is the potential outcome for that same individual if allocated into the control group. Both potential outcomes cannot be observed simultaneously for an individual. The ATE, represented by *τ*, is calculated by averaging across all patients in the study.

In observational studies where treatment group allocation is not random, there can be imbalances in covariate distributions between the treatment and control groups. Using traditional statistical (non-causal) methods that just compare means between these groups, often through statistical tests, can lead to biased estimates of treatment effects [7, 8, 10]. These biases often originate from *unadjusted* *covariates* or confounders, which are factors influencing both the treatment decision and the outcome. These confounders can create misleading (or spurious) connections between the treatment and its effects [5, 11, 12]. In our analysis, the covariate variable *X* is regarded as a representative confounder that influences both the treatment variable *W* and the outcome *Y*. We note that *X* encompasses a range of individual characteristics, each potentially acting as a confounder. For instance, the number of work hours, as a component of *X*, could influence both the amount of exercise a person engages in (treatment) and their BMI level (outcome). Individuals with demanding work schedules might have less time for exercise, but could still exhibit a lower BMI due to the physical demands of prolonged working hours. This scenario can obscure the actual causal relationship between exercise and BMI levels.

To account for the influence of *X*, Augmented Inverse Propensity Weighting (AIPW) has been proposed as a method to estimate the ATE. In line with the standard assumptions for causal inference, namely consistency, ignorability, and positivity, AIPW calculates the ATE by combining an outcome model $\left(\hat{\mu }\left(X,w\right)=E\left[Y|X,W=w\right]\right)$ and a propensity model $\left(\hat{p}\left(X\right)=P\left(W=1|X\right)\right)$. The specific formula used in AIPW for calculating ATE is detailed in Eq 4 below:

$$\begin{array}{c} \begin{array}{cc} {\hat{\tau }}^{\text{AIPW}} & ≔\frac{1}{n}\sum_{i=1}^{n}[{\hat{\mu }}^{-i}\left(X_{i},1\right)-{\hat{\mu }}^{-i}\left(X_{i},0\right)]\\ & +\frac{W_{i}}{{\hat{p}}^{-i}\left(X_{i}\right)}[Y_{i}-{\hat{\mu }}^{-i}\left(X_{i},1\right)]-\frac{1-W_{i}}{1-{\hat{p}}^{-i}\left(X_{i}\right)}[Y_{i}-{\hat{\mu }}^{-i}\left(X_{i},0\right)] \end{array} \end{array}$$ (4)

A key advantage of AIPW is its ability to produce an unbiased and consistent ATE estimate, provided that at least one of the models—either the outcome or the propensity model—is accurate and consistent. To ensure this, cross-fitting is used in the estimation process, where predictions for an individual *i* are based on models trained without including that individual’s data, thus mitigating the risk of overfitting. This approach serves as the rationale behind the “-i” superscript in our formula 4. In our study, the outcome and propensity models are trained using non-parametric random trees and forests. We discuss the advantages of using these methods in the following sections.

Importantly, AIPW-adjusted values are intended for aggregate use, such as in estimating the ATE across a population, rather than representing treatment effects at an individual level. It is also important to note that the method assumes positivity. This means the values of $\hat{p}\left(X\right)$ should not be extremely close to 0 or 1, as such extremes can lead to less reliable estimates. In our observational data, this positivity assumption holds true and has been rigorously tested using our experimental health data.

### 3.2 Heterogeneity in Treatment Effects (HTE)

Typically, the impact of a treatment varies across a population, with factors like sex, age, and occupation influencing individual responses. This variability, known as heterogeneity, implies that the ATE, which measures the overall effect across the entire population, does not fully capture the nuances of treatment effects within different sub-populations or how these effects might shift in a population with distinct characteristics. Adding to this, understanding and interpreting treatment effects in specific sub-groups is crucial for comprehending the data more thoroughly and, consequently, aids in strategising more effective treatments tailored to these sub-groups.

When we are interested in assessing heterogeneity of treatment effects across different subgroups within the population, we focus on estimating the Conditional Average Treatment Effects (CATE). CATE is specifically calculated for each subgroup, denoted as *S*, and is defined as follows:

$$\begin{array}{c} \tau \left(S\right)≔E[Y(1)-Y(0)|X\in S] \end{array}$$ (5)

#### 3.2.1 Pre-specified subgroups

In our analysis, we first deal with subgroups that are defined before examining the data, a scenario we refer to as pre-specified subgroups. For instance, in some studies, patients might be categorised into age-based groups like ‘young’ and ‘old’ as part of the research design. In this context, our focus is on estimating the CATE for each of these predetermined subgroups for all individuals. The contrasting approach, where subgroups are determined based on patterns observed in the data, is explored in the subsequent section of our study.

Let *G* be the categorical variable indicating which group an individual belongs to. The CATE for each specific group, denoted as (*G*_*i*_ = *g*_*i*_), is defined below:

$$\begin{array}{c} \tau \left(g\right)≔E[Y\left(1\right)-Y\left(0\right)|G_{i}=g_{i}] \end{array}$$ (6)

Using these CATE estimates, we are also able to perform hypothesis tests to evaluate the variance in the treatment effect’s magnitude between different groups. When dealing with more than two groups, it is possible to conduct pairwise comparisons to assess differences:

$$\begin{array}{c} \begin{array}{cc} H_{0} & :E[Y(1)-Y(0)|G=g]=E[Y\left(1\right)-Y\left(0\right)|G=g^{\prime }]\\ & \text{for}\;\text{all}\;\text{pairs}\;\text{of}\;\text{group}(g,g^{\prime }) \end{array} \end{array}$$ (7)

In scenarios with multiple group comparisons, adjusting the resulting p-values is necessary to avoid error inflation in hypothesis tests. To address the issue of multiple comparisons, methods like the Bonferroni correction and the Romano-Wolf correction can be employed [66]. These techniques adjust the p-values to lower the risk of false discoveries, effectively managing the problem of multiple comparisons.

In the simplest case where the population is divided into two mutually exclusive subsets, denoted as *G* ∈ {0, 1}, we can formulate and test the following hypothesis:

$$\begin{array}{c} H_{0}:\;E[Y(1)-Y(0)|G=1]=E[Y(1)-Y(0)|G=0] \end{array}$$ (8)

#### 3.2.2 Data-driven subgroups

In cases where groups are not predetermined, it becomes valuable to derive subgroups based on data, grouping individuals such that each subgroup has a relatively uniform response to the treatment. This data-driven approach can uncover important classifications that are key to predicting how an individual might respond to the treatment. To achieve this, we utilise the causal tree framework, which is effective in identifying such subgroups and estimating the variability in treatment effects across them.

In considering randomised data, building a causal tree typically involves “*balancing*” in-group heterogeneity with tree complexity. The learning algorithm aims to group individuals based on covariate values, minimising the uncertainty variation in the CATE for each group. However, this process might lead to groups with only a single individual, making it necessary to consider the tree’s complexity—such as its depth or number of leaves—to ensure it’s both meaningful and practical. In Athey and Imbens’ implementation of causal trees [42], a crucial “*balance*” parameter is the minimum required number of individuals in each group. If a group identified by the algorithm is smaller than this threshold, it will not form a separate leaf but will merge with others instead. Setting this parameter is critical: if it is equal to the total number of observations, the tree will have just one leaf, whereas if it is too low, the tree might yield limited useful insights.

Once the causal tree is constructed, it is also recommended to verify the heterogeneity of treatment effects between the different tree leaves. This verification can be performed using pairwise comparisons through a series of two-sample tests. In these tests, we examine whether there is a significant difference in the treatment effect between each possible pair of leaves. Intuitively, the sub-groups identified should display significant differences in treatment effects, while individuals within a single sub-group are expected to have a relatively consistent response to the treatment.

It is important to recognise that the causal tree framework was initially designed for randomised data, in which covariates do not impact treatment assignment. Therefore, in observational studies where covariates significantly influence treatment allocation, we use AIPW to reweight individual observations and correct for confounding factors. Specifically, before passing the data into the causal tree learning algorithm, the outcome is transformed/reweighted in a slightly similar manner as Eq 4 as follows:

$$\begin{array}{cc} Y_{i}^{\text{AIPW}} & ≔W_{i}[{\hat{\mu }}^{-i}\left(X_{i},1\right)+\frac{1}{{\hat{p}}^{-i}\left(X_{i}\right)}(Y_{i}-{\hat{\mu }}^{-i}\left(X_{i},1\right))]\\ & +(1-W_{i})[{\hat{\mu }}^{-i}\left(X_{i},0\right)+\frac{1}{1-{\hat{p}}^{-i}\left(X_{i}\right)}(Y_{i}-{\hat{\mu }}^{-i}\left(X_{i},0\right))] \end{array}$$ (9)

Subsequently, *Y*^AIPW^ is utilised as the outcome variable in constructing the tree, treating as though it were derived from a genuinely randomised study. Causal trees segment populations into subgroups based on covariates, offering clear rules and maintaining predictive accuracy, aiding practitioners in treatment targeting as detailed in the next section. However, their variability due to different data splits impacts subgroup treatment effect estimation reproducibility. To address this, causal forests, comprising multiple causal trees each using unique observation and covariate subsets, are employed, enhancing result consistency as detailed in Section 2.

### 3.3 Intervention targeting strategies

Given the testable hypothesis that different subgroups within a population will react differently to a treatment, a crucial subsequent question arises: how can a treatment be most effectively targeted within a population for optimal impact? For health practitioners and researchers, answering this question is key to strategising the best policies for patient care and maximising overall population health outcomes.

Utilising causal forests, we can explore the ATE estimates for individuals characterised by specific observable traits (covariates), under varying treatment conditions. The interpretability of causal trees within these forests is also a significant advantage, as it enhances our understanding of how different subgroups respond to treatment. This understanding is instrumental in optimising resource allocation and improving overall population health outcomes, particularly in situations where not all individuals can or should receive treatment. By applying this methodology, we can effectively evaluate and compare different intervention targeting strategies, assessing their impact on overall population health.

We can represent the intervention targeting strategy as a function *π*(*x*) ∈ [0; 1]. When (*π*(*x*) = 1), it indicates that individuals with covariates (*X* = *x*) receive treatment, while (*π*(*x*) = 0) means they are not treated. Regarding the intervention targeting strategy *π*(*x*), two essential questions arise:

- What is the expected outcome when individuals are assigned treatment based on this strategy, represented as $E[Y_{i}(\pi (X_{i}))]$?
- Is *π*(*x*) superior to another strategy *π*′(*x*)? In other words, when larger outcomes indicate the treatment’s benefit, is $E[Y(\pi (X))]>E[Y(\pi^{\prime }\left(X\right))]$?

It is worth noting that the average treatment effect (ATE) corresponds to a specific scenario of comparing two strategies: one where everyone receives treatment (*π*(*x*) ≡ 1), and the other where no one receives treatment (*π*′(*x*) ≡ 0).

To address these questions, we once again make use of the AIPW scores. To estimate the expected outcome of a strategy *π*(*x*), we calculate the average of individual AIPW scores using the following formula:

$$\begin{array}{c} E[Y(\pi (X))]\approx \frac{1}{n}\sum_{i=1}^{n}{\hat{\Gamma }}_{i,\pi (X_{i})}, \end{array}$$ (10)

where ${\hat{\Gamma }}_{i,\pi (X_{i})}=\pi \left(X_{i}\right)\cdot {\hat{\Gamma }}_{i,1}+(1-\pi (X_{i}))\cdot {\hat{\Gamma }}_{i,0}$ is the estimated outcome of individual *i* under treatment strategy *π*. Here, ${\hat{\Gamma }}_{i,0}$ and ${\hat{\Gamma }}_{i,1}$ represent the estimated outcomes for individual *i*, if they were untreated or treated, respectively.

Therefore, the difference between two intervention targeting strategies *π* and *π*′ can be estimated as follows:

$$\begin{array}{c} E[Y(\pi (X))-Y(\pi^{\prime }\left(X\right))]\approx \frac{1}{n}\sum_{i=1}^{n}({\hat{\Gamma }}_{i,\pi (X_{i})}-{\hat{\Gamma }}_{i,\pi^{\prime }\left(X_{i}\right)}) \end{array}$$ (11)

#### 3.3.1 Non-parametric strategies

After obtaining CATE estimates, one initial strategy is to treat only those groups that benefit from the treatment, leaving everyone else untreated. This strategy, known as a non-parametric strategy, if a positive response is indicated by $\left(\hat{\tau }\left(x\right)>0\right)$, can be translated as follows:

$$\begin{array}{c} \pi^{*}\left(x\right)=\{\begin{array}{cc} 1 & \text{if}\;\hat{\tau }\left(x\right)\geq 0\\ 0 & \text{otherwise} \end{array} \end{array}$$ (12)

To ensure the fairness of the strategy value estimate, it is important that $\hat{\tau }\left(x\right)$ is estimated using a distinct dataset that does not overlap with the one used for estimating π*(*x*) and $E[Y(\pi^{*}\left(x\right))]$.

#### 3.3.2 Parametric strategies

The non-parametric intervention targeting strategy mentioned previously has a limitation in that it may be challenging to clearly define the characteristics that distinguish individuals in the treatment group from those who are untreated. In other words, it can be challenging to predict in advance who will benefit from the intervention (a *priori*) based solely on their covariate information.

For the purpose of rolling out a program in a community, parametric strategies derived from tree models offer valuable insights. These strategies enable a clear segmentation of the population based on their covariates, providing a *transparent* and *interpretable* approach to intervention targeting. Further, using this method, a researcher can determine the level of specificity used to divide individuals into treatment and control groups, which is controlled by the depth of the tree.

When a specific tree depth is chosen, the method proceeds by computing AIPW scores for each individual, which represent their predicted adjusted outcomes under both treatment and control conditions. The data is then divided into two sets: a training set and a test set. In the training set, a tree is constructed, representing the optimal targeting strategy. Subsequently, estimates of the outcomes for each subgroup (corresponding to the leaves of the tree and represented for specific individual’s covariates) are calculated using the test set.

It is worth noting that, within our framework, both non-parametric and parametric intervention targeting strategies utilise causal trees/forests. However, non-parametric strategies rely on the estimated CATE, while parametric strategies are based on the individuals’ covariates.

### 3.4 Time complexity

The training time complexity of our framework is determined by the number of individual causal trees *B* in the causal forest, as well as the time required to construct each causal tree. On average, we assume the causal tree is balanced, meaning the final tree depth is proportional to log(*n*). Building the tree involves three steps: i) finding the best split at each node, which costs *O*(*d* × *n* log *n*) since we iterate and sort each feature’s values across *n* samples; ii) performing splits across *O*(*n*) nodes. Thus, the overall complexity of building the tree is *O*(*d* × *n*^2^ log *n*). Given that the causal forest consists of *B* trees, the total complexity of the framework is *O*(*B* × *d* × *n*^2^ log *n*). We note that as *B* increases, the framework becomes more robust because each tree is trained on a different subsample of the data, similar to cross-validation. The prediction of causal effects is then averaged across the *B* trees, leading to more reliable and accurate estimates. At inference, the time complexity of our framework is *O*(*B* log *n*) because, for each test sample, we traverse the maximum depth of each causal tree, which is *O*(log *n*).

### 3.5 Ethics statement

This study uses existing de-identified data from the National Health Survey, 2017–18, accessed in accordance with the Australian Bureau of Statistics’ agreements. All data were collected with informed consent, and privacy safeguards were applied following ABS ethical standards.

## 4 Demonstration

### 4.1 Data

The Australian National Health Survey is conducted every three years and seeks to establish a nationally representative cross-sectional profile of the country’s health and wellbeing. The results from the 2017–18 survey were used in this study, comprising interview-style responses from 21,315 individuals (both adults and children) [22]. Our study specifically focuses on adults aged 18 and older. After rigorously filtering the dataset to exclude any instances with missing data in any of the covariates, outcomes, or exposure variables, our analysis retained a total of 9,052 individuals. Of these, 38% were classified as having overall moderate to high levels of exercise, based on responses to multiple questions about their physical activities in the last week. Our outcome is measured BMI and the causal question of interest is whether BMI is affected by moderate to high levels of exercise (treatment), compared to low to no exercise (control). Table 1 details the covariates used in the analysis.

**Table 1:** Trial covariates and response.

Variable	Description	Type
Age Group	Age group classification	Factor: Age in years divided to 15 groups
Sex	Male / Female	Binary
Income Decile	Income ranked in deciles	Factor: 10 deciles
Working Time	Working time per week	Factor: Working hours divided to 7 groups
Education Level	Level of education	Factor: 4 levels of education from lowest to highest
Socio-economic Status Decile	Socio-economic index decile	Factor: 10 deciles
Remoteness	Household location—remoteness category	Factor: 3 levels of remoteness
Fruit and vegetable Consumption	Met fruit and/or vegetable consumption guideline or not	Binary
Sweet drink Consumption	Number of cups of selected sugary drinks consumed per week	Integer
BMI	Measured BMI (kg/m^2^)	Continuous (**response**)
Exercise Level	Treatment = {moderate to high}. Control = {Low to No exercises}	Binary (**treatment**)

Prior to proceeding with the causal queries of interest, we first verify the testable assumption needed for the use of our framework—the *overlap* or *positivity* assumption mentioned in Section 2. The result is reported in S1 Appendix, confirming that this assumption is satisfied within the dataset under consideration.

### 4.2 Average treatment effect

We estimate the ATE (Average Treatment Effect across the entire population) using two different methods and then compare the results. The first method we use is the difference in means estimator, typically calculated using traditional statistical or non-causal methods. This estimator [E(Y∣W=1)-E(Y∣W=0)] represents the difference in the average outcome between individuals with moderate/high levels of exercise (*W* = 1) and those with low levels of exercise (*W* = 0). The second method we utilise is the causal forest-based AIPW adjusted estimator, as outlined in Eq 4. This estimator allows us to estimate the causal treatment effect while taking into account potential differences in covariate distributions between the two groups. The estimated ATE values, with 95% CI were (−1.62 ± 0.19) for the first method and (−1.13 ± 0.19) for the second method, respectively.

While the causal forest-based AIPW estimate suggests that moderate to high levels of exercise result in a smaller reduction in BMI compared to the non-causal methods, it is important to note that the non-causal methods may overestimate this reduction. This overestimation can occur due to biases stemming from the unbalanced distribution of covariates between the treatment and control groups, which can ultimately impact the accuracy of the estimates.

**Fig 3:**
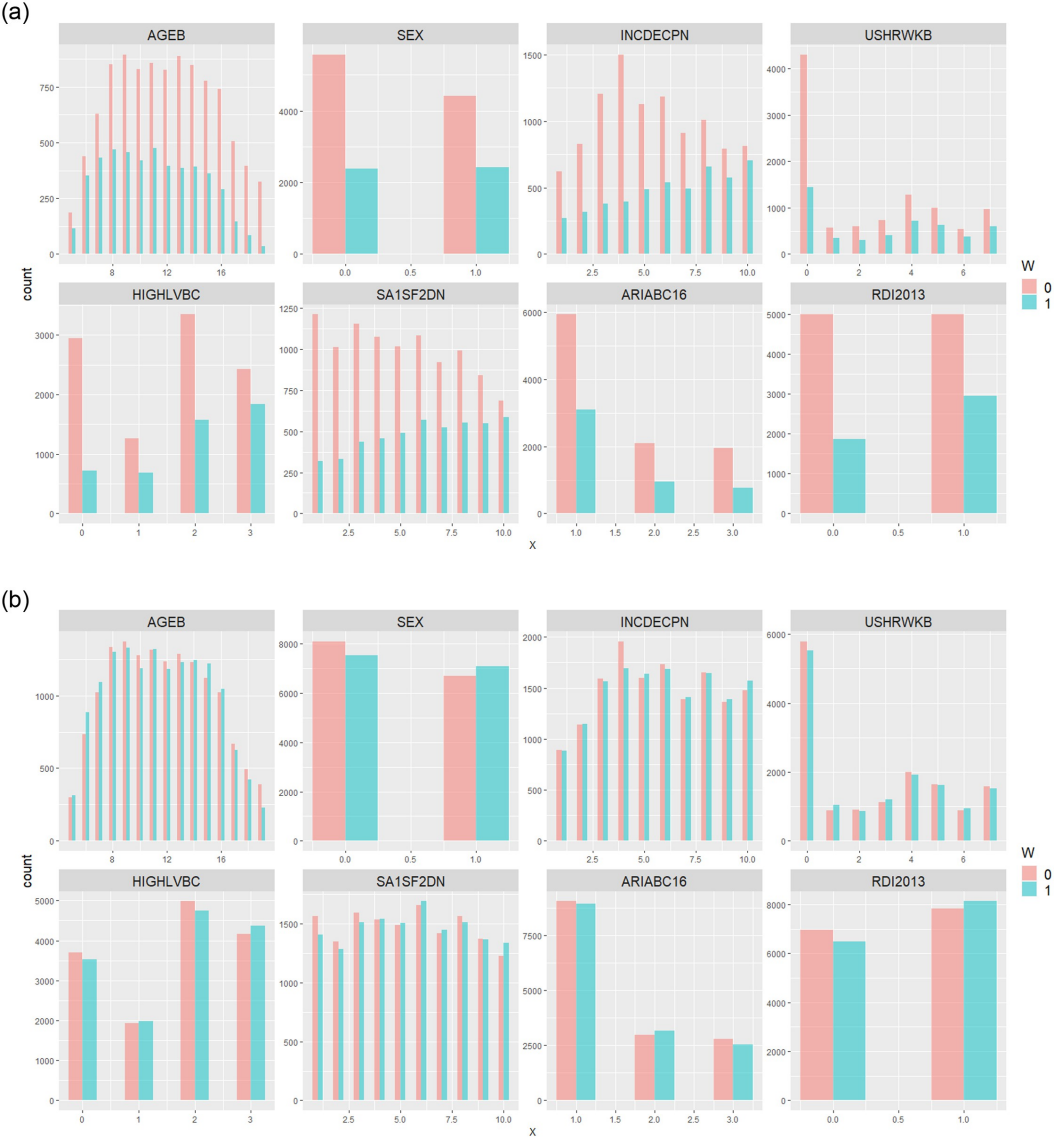
Comparison of Covariate Distributions: (a) Before and (b) After AIPW Adjustment. Prior to AIPW adjustment, certain covariates exhibit disparities in distribution between the control (*W* = 0) and treatment (*W* = 1) groups, resulting in confounding bias. Following AIPW adjustment, individual responses are weighted by the IPW score, resulting in a more balanced representation of covariates between the treated and control groups. Here, **INCDEPN** represents gross weekly *personal income* in deciles, **USHRWKB** denotes the *hours* usually *worked* each week across all jobs, and **HIGHLVBC** indicates the level of highest *educational attainment*. **SA1SF2DN** stands for the index of relative *socio-economic disadvantage* in 2016, at the SA1 level, presented in deciles nationally. **ARIABC16** is used for *remoteness* area categories, reported in 2016. **RDI2013** signifies adherence to the recommended *vegetable and fruit consumption* as per the 2013 NHRMC guidelines. (**a**) Covariate Distribution for treatment (*W* = 1) and untreated (*W* = 0) groups, before AIPW adjustment. (**b**) Covariate Distribution for treatment (*W* = 1) and untreated (*W* = 0) groups, after AIPW adjustment.

We further investigate the distribution of covariates between the treatment and control groups both before and after applying AIPW adjustments. Fig 3 illustrates the covariate distributions before (Fig 3a) and after (Fig 3b) AIPW adjustment. Prior to adjustment, we can see that there is an imbalance in the distribution of covariates between the two groups, indicating the presence of potential confounding bias in the difference-in-means (non-causal) estimator. This bias can lead to inaccurate and unreliable estimates. However, following the implementation of AIPW adjustments, individual responses are carefully weighted, resulting in a more balanced representation of covariates between the treated and control groups. With this improved balance, any differences in outcomes between the two groups are more likely to be attributed to the treatment itself rather than confounding covariates. This indicates a substantial reduction in confounding bias, particularly for the covariates included in the adjustment.

### 4.3 Heterogeneity in treatment effect

#### 4.3.1 Heterogeneity detection

Before delving into a more in-depth analysis aimed at identifying subgroups with a heterogeneous treatment effect response, it is important to validate whether our learnt causal forest is accurately capturing any heterogeneity in treatment effect size across individuals present in the data. To assess this, we employ a linear predictor analysis to test for the presence of significant heterogeneity [67], as described detailed in S1 Appendix. The results of this test confirm the existence of treatment effect heterogeneity with high statistical significance, indicating that our model effectively captures this variation.

#### 4.3.2 HTE: Pre-specified sub-groups

To assess whether there are significant variations in treatment effects among subgroups determined by common factors such as gender or education level, we perform hypothesis tests to examine differences between these subgroups.

In S1 Appendix, we provide the results of hypothesis tests conducted by dividing the population into *two* groups based on gender and *four* groups based on education level. These tests reveal a significant disparity in BMI reduction attributed to exercise between males and females, with a difference of 1.031 units. However, the BMI reduction associated with exercise remains relatively consistent across different education levels.

#### 4.3.3 HTE: Data-driven sub-groups

**Fig 4:**
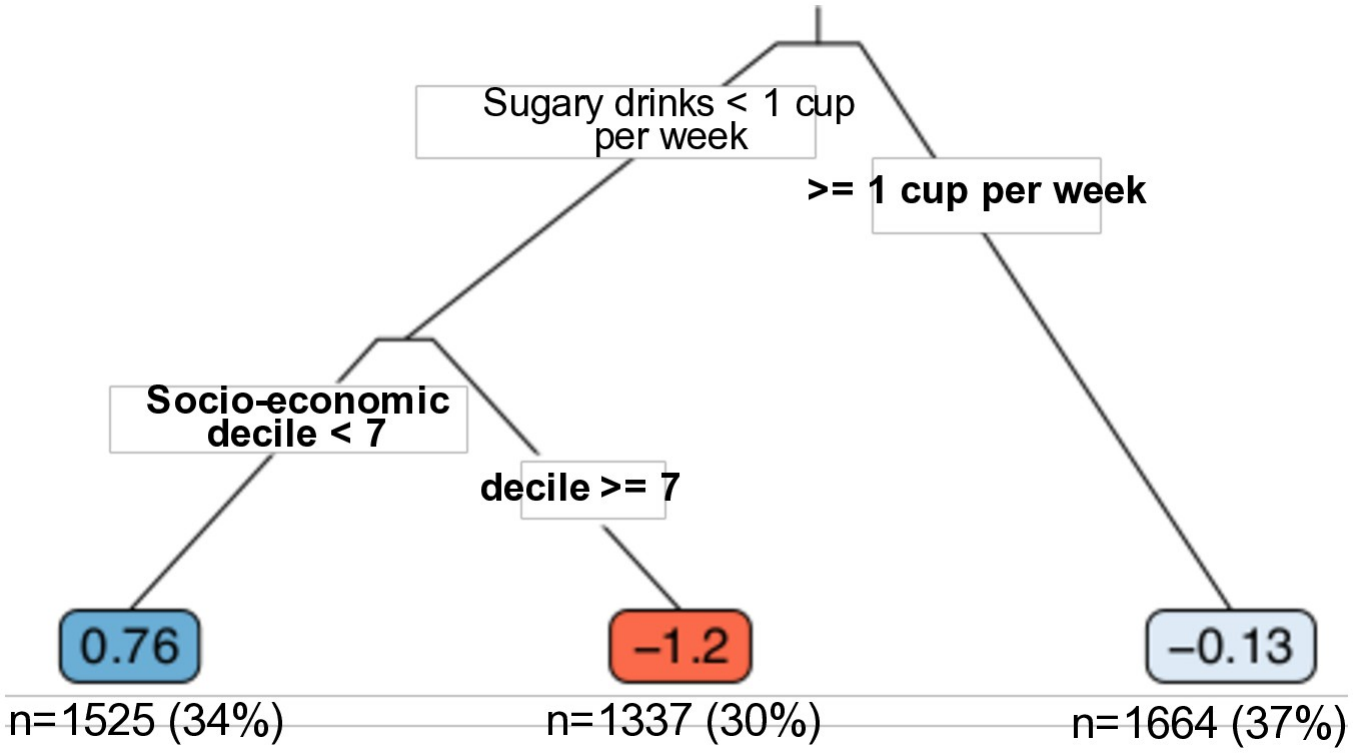
The causal tree learnt from the National Health Survey data. The numbers in each leaf of the tree represent the CATE calculated for the observations within that leaf. The tree’s findings suggest that individuals in the 7th to 10th deciles of the socio-economic index who consume less than one cup of selected sugar-sweetened beverages per week experience a significant BMI reduction of 1.2 units when engaging in moderate to high levels of exercise. This effect is in contrast to individuals with a similar covariate profile who do not exercise at this intensity. For those who consume sugar, the BMI reduction is notably smaller, at only 0.13 units.

To examine heterogeneous treatment effects automatically discovered in a data-driven manner, in Fig 4 we illustrate one example of a causal tree generated from the data set. The tree categorises the population into three approximately equal groups, each corresponding to one of its three leaves. Interestingly, individuals in the 1st to 6th deciles of the socio-economic index who do not consume sugary drinks exhibit a slightly higher BMI (by 0.176 units) when exposed to the treatment (exercise). In contrast, the response for the rest of the population goes in the expected direction, with a lower BMI observed in those who engage in exercise.

To further investigate the differences between subgroups divided by the causal tree, in Table 2 we perform comparisons comparisons between each leaf and the second leaf, which exhibits the most pronounced response to treatment. The findings indicate a significant disparity between the furthest leaves, with an over 2-unit difference (*p* = 0.036) in average treatment effects between the second and first leaves.

**Table 2:** Testing variations in treatment effects among different leaves of the causal tree for the National Health Survey data. The adjusted *p*-values are obtained using the Romano-Wolf correction [66].

	Difference	Standard Error	Original *p*-value	Adjusted *p*-value
Leaf 1 v.s. Leaf 2	2.009	0.858	0.019	0.036
Leaf 3 v.s. Leaf 2	1.121	0.857	0.191	0.183

### 4.4 Optimal intervention strategy learning

#### 4.4.1 Optimal non-parametric strategies

We first examine two straightforward non-parametric intervention targeting strategies. Initially, we only give treatment to those who were predicted to have BMI level decreased by the treatment. More specifically, we treat those who have negative predicted conditional average treatment effects. The strategy, which is denoted as *π*^−^, is then specified as follows:

$$\begin{array}{c} \pi^{-}\left(x\right)=\{\begin{array}{cc} 1 & \text{if}\;\hat{\tau }\left(x\right)<0\\ 0 & \text{otherwise} \end{array} \end{array}$$ (13)

**Fig 5:**
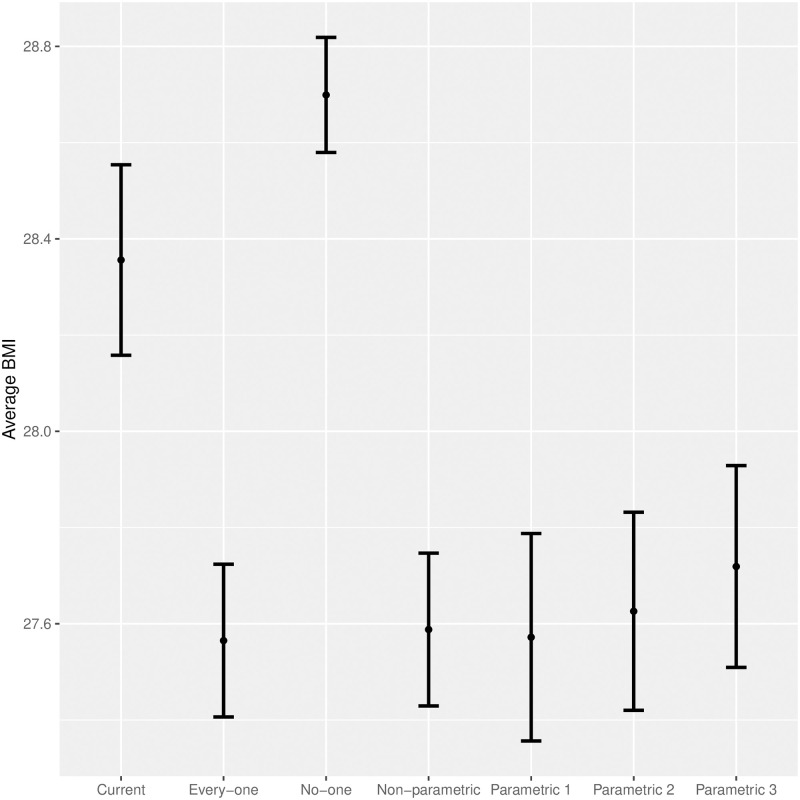
Comparing the impact of optimal parametric and non-parametric policies with “no treatment” and “universal treatment”.

Additionally, we compare the value of the aforementioned strategy with a random targeting strategy that assigns an individual to treatment or control groups with equal probability (*π*′(*x*) ≡ 0.5). The obtained results follow a natural intuition: targeting treatment exclusively to those anticipated to experience a reduction in BMI under the treatment indeed *lowers* the average BMI to 27.205±0.116, compared with the randomised treatment strategy whose average BMI of 27.764 ± 0.083 (Fig 5).

#### 4.4.2 Optimal parametric strategies

We can use a policy tree to develop more intricate yet interpretable intervention strategies, basing the strategy decisions on the individual characteristics of the individuals. We developed three tree architectures of depths one, two, and three. These architectures can be found in S1 Appendix. We can now compare the average BMI in the population as a result of these parametric strategies (Fig 5). This can be compared against: (1) the current treatment/control assignment in the data set, (2) treating everyone, (3) having everyone be in control, and (4) the non-parametric policy (i.e., the aforementioned *π*^−^ strategy). These results are shown in Fig 5 with the percentage of individuals treated under each strategy shown in Table 3.

**Table 3:** Percentage of individuals treated within each treatment/control strategy.

Current	Non-para	Para 1	Para 2	Para 3
32.5	95.9	98.0	93.9	83.5

### 4.5 Discussion

#### 4.5.1 Method scalability

Our causal forest framework for estimating outcomes and propensity scores is inherently scalable, expanding with larger datasets by growing more trees and leaves, leading to increasingly accurate and reliable estimates. Previous theoretical work [15] shows that, with properly chosen subsample sizes for each causal tree, causal forest estimates remain asymptotically Gaussian and *unbiased*, even in large datasets. Unlike traditional epidemiological methods that adjust for one confounder at a time, our approach simultaneously handles multiple confounders, making it highly effective for large-scale data.

#### 4.5.2 Limitation

In addition to the testable overlap or positivity assumption, which we have validated, our framework assumes no latent variables or hidden confounders, meaning the covariates in the dataset are sufficient to adjust for confounders and accurately estimate treatment effects. By controlling for all these covariates, our framework aims to provide reliable causal effect estimates while also capturing heterogeneity across subgroups. While our approach is robust for the available data, challenges may arise if latent variables are present in the dataset [60, 62]. Handling unobserved confounders requires more advanced methods, which we leave for future work.

## 5 Conclusion and future work

In this study, we demonstrate the application of machine learning-based causal inference techniques, specifically causal trees and forests, to address simulated causal queries in the domain of health research. By employing data from the 2017–18 Australian National Health Survey, we demonstrate how these methods can effectively answer questions about the impact of exercise on BMI levels. This includes the assessment of the average treatment effect, the examination of treatment effect variations across different subgroups, and the identification of optimal intervention targeting strategies. This is attributed to the inherent strengths of causal trees and forests, notably their robust capacity for estimating causal effects and their clarity in interpretability. The wide-ranging applicability of these methods to a variety of critical questions underscores their significant potential for uncovering insights into causal relationships, evaluating the efficacy of interventions, and optimising resource distribution in the field of public health intervention research.

## Supporting information

S1 Appendix This appendix details how our method addresses confounding variables, presents supplementary experimental results, and includes overlap assumption tests, heterogeneity analyses of treatment effects, pre-specified subgroup evaluations in HTE, covariate analyses, cohort rankings, and optimal parametric strategies, with accompanying tables presenting additional results for each section. (PDF)

S1 FigHistogram of the propensity score $\hat{p}(x)$.This is used to assess the overlap assumption in our method.(JPG)

S2 Fig Correlation between all variables. (JPG)

S3 Fig Individuals ranked into five equally sized cohorts based on treatment effect. Q1 is largest response and Q5 is smallest response.
(JPG)

S4 Fig Average covariate values within five cohorts based on CATE estimated ranking. Q1 is 20% of data with the most significant treatment effect, while Q5 is the 20% of data with the worst improvement in treatment effect.
(PDF)

S5 Fig 95% Confidence interval estimates of average treatment effect over the range of age groups. All other covariates at kept at their median value.
(JPG)

S6 Fig Predicted average treatment effect over the range of Age groups for each Sex level (Male = 1 and Female = 0). Standard error provided in brackets. All other covariates at kept at their median value.
(JPG)

S7 Fig Tree depth = 1: Optimal parametric intervention targeting strategy, with left arrows indicating “True” and right arrows indicating “False”. Decision conditions at this depth is if individuals are in Age Group less than or equal to 5 (these are individuals aged 19 or less).
(PNG)

S8 Fig Tree depth = 2: Optimal parametric intervention targeting strategy, with left arrows indicating “True” and right arrows indicating “False”. Decision conditions at this depth include whether individuals responded to the Fruit and Vegetable consumption guideline, are in the lowest Socio-economic decile (Socio-economic disadvantage index), and if in Age Group less than or equal to 5 (individuals aged 19 or less).
(PNG)

S9 Fig Tree depth = 3: Optimal parametric intervention targeting strategy, with left arrows indicating “True” and right arrows indicating “False”. Decision conditions at this depth include whether Sugar Consumption (Weekly cups of sugar sweetened drinks) is less than equal to 0 or 2, Fiber Consumption (Fruit and Vegetable consumption guideline) was responded to, if in Age Group less than or equal to 5 (aged 19 or less) or 8 (aged 34 or less), and Working Time (Hours usually worked per week) is less than or equal to 24 hours.
(PNG)

## References

[1] Pearl J. Causal inference in the health sciences: a conceptual introduction. Health services and outcomes research methodology. 2001;2:189–220. doi: 10.1023/A:1020315127304

[2] Poghosyan H, Sheldon LK, Leveille SG, Cooley ME. Health-related quality of life after surgical treatment in patients with non-small cell lung cancer: a systematic review. Lung cancer. 2013;81(1):11–26. doi: 10.1016/j.lungcan.2013.03.013 23562675

[3] Burroughs VJ, Maxey RW, Levy RA. Racial and ethnic differences in response to medicines: towards individualized pharmaceutical treatment. Journal of the National Medical Association. 2002;94(10 Suppl):1. 12401060 PMC2594139

[4] Kabisch M, Ruckes C, Seibert-Grafe M, Blettner M. Randomized Controlled Trials. Dtsch Arztebl International. 2011;108(39):663–668. doi: 10.3238/arztebl.2011.0663 22013494 PMC3196997

[5] Pearl J. Causality: Models, Reasoning and Inference. Cambridge University Press; 2009.

[6] Stanley K. Design of randomized controlled trials. Circulation. 2007;115(9):1164–1169. doi: 10.1161/CIRCULATIONAHA.105.594945 17339574

[7] Vapnik V. The nature of statistical learning theory. Springer science & business media; 1999.

[8] Sterne JA, Gavaghan D, Egger M. Publication and related bias in meta-analysis: power of statistical tests and prevalence in the literature. Journal of clinical epidemiology. 2000;53(11):1119–1129. doi: 10.1016/S0895-4356(00)00242-0 11106885

[9] Peters J, Janzing D, Schölkopf B. Elements of causal inference: foundations and learning algorithms. The MIT Press; 2017.

[10] Schölkopf B. Causality for machine learning. In: Probabilistic and Causal Inference: The Works of Judea Pearl; 2022. p. 765–804.

[11] Streeter AJ, Lin NX, Crathorne L, Haasova M, Hyde C, Melzer D, et al. Adjusting for unmeasured confounding in nonrandomized longitudinal studies: a methodological review. Journal of clinical epidemiology. 2017;87:23–34. doi: 10.1016/j.jclinepi.2017.04.022 28460857 PMC5589113

[12] Kahlert J, Gribsholt SB, Gammelager H, Dekkers OM, Luta G. Control of confounding in the analysis phase–an overview for clinicians. Clinical epidemiology. 2017; p. 195–204. doi: 10.2147/CLEP.S129886 28408854 PMC5384727

[13] Burkhart MC, Ruiz G. Neuroevolutionary representations for learning heterogeneous treatment effects. Journal of Computational Science. 2023;71:102054. doi: 10.1016/j.jocs.2023.102054

[14] Künzel SR, Sekhon JS, Bickel PJ, Yu B. Metalearners for estimating heterogeneous treatment effects using machine learning. Proceedings of the national academy of sciences. 2019;116(10):4156–4165. doi: 10.1073/pnas.1804597116 30770453 PMC6410831

[15] Wager S, Athey S. Estimation and Inference of Heterogeneous Treatment Effects using Random Forests. Journal of the American Statistical Association. 2018;113(523):1228–1242. doi: 10.1080/01621459.2017.1319839

[16] Gillman MW, Hammond RA. Precision Treatment and Precision Prevention: Integrating “Below and Above the Skin”. JAMA Pediatrics. 2016;170(1):9–10. doi: 10.1001/jamapediatrics.2015.2786 26595371 PMC4705446

[17] Imai K, Keele L, Tingley D. A general approach to causal mediation analysis. Psychological methods. 2010;15(4):309. doi: 10.1037/a0020761 20954780

[18] Listl S, Jürges H, Watt RG. Causal inference from observational data. Community dentistry and oral epidemiology. 2016;44(5):409–415. doi: 10.1111/cdoe.12231 27111146

[19] Hammerton G, Munafò MR. Causal inference with observational data: the need for triangulation of evidence. Psychological medicine. 2021;51(4):563–578. doi: 10.1017/S0033291720005127 33682654 PMC8020490

[20] Nichols A. Causal inference with observational data. The Stata Journal. 2007;7(4):507–541. doi: 10.1177/1536867X0800700403

[21] Strobl EV, Lasko TA. Identifying patient-specific root causes with the heteroscedastic noise model. Journal of Computational Science. 2023;72:102099. doi: 10.1016/j.jocs.2023.102099

[22] Australian Bureau of Statistics. National Health Survey: First Results, 2017-18 financial year; 2028.

[23] Indyk P, Motwani R. Approximate nearest neighbors: towards removing the curse of dimensionality. In: Proceedings of the thirtieth annual ACM symposium on Theory of computing; 1998. p. 604–613.

[24] Taghikhah F, Voinov A, Filatova T, Polhill JG. Machine-assisted agent-based modeling: Opening the black box. Journal of Computational Science. 2022;64:101854. doi: 10.1016/j.jocs.2022.101854

[25] Parthiban G, Srivatsa S. Applying machine learning methods in diagnosing heart disease for diabetic patients. International Journal of Applied Information Systems. 2012;3(7):25–30. doi: 10.5120/ijais12-450593

[26] Otoom AF, Abdallah EE, Kilani Y, Kefaye A, Ashour M. Effective diagnosis and monitoring of heart disease. International Journal of Software Engineering and Its Applications. 2015;9(1):143–156.

[27] Christmas J, Keedwell E, Frayling TM, Perry JR. Ant colony optimisation to identify genetic variant association with type 2 diabetes. Information Sciences. 2011;181(9):1609–1622. doi: 10.1016/j.ins.2010.12.005

[28] Zhang B, Karray F, Li Q, Zhang L. Sparse representation classifier for microaneurysm detection and retinal blood vessel extraction. Information Sciences. 2012;200:78–90. doi: 10.1016/j.ins.2012.03.003

[29] Sen SK, Dash S. Application of meta learning algorithms for the prediction of diabetes disease. International Journal of Advance Research in Computer Science and Management Studies. 2014;2(12).

[30] Iyer A, Jeyalatha S, Sumbaly R. Diagnosis of diabetes using classification mining techniques. arXiv preprint arXiv:150203774. 2015;.

[31] Li DC, Fang YH, Lai YY, Hu SC. Utilization of virtual samples to facilitate cancer identification for DNA microarray data in the early stages of an investigation. Information Sciences. 2009;179(16):2740–2753. doi: 10.1016/j.ins.2009.04.003

[32] Senturk ZK, Kara R. Breast cancer diagnosis via data mining: performance analysis of seven different algorithms. Computer Science & Engineering. 2014;4(1):35.

[33] Majali J, Niranjan R, Phatak V, Tadakhe O. Data mining techniques for diagnosis and prognosis of cancer. International Journal of Advanced Research in Computer and Communication Engineering. 2015;4(3):613–616. doi: 10.17148/IJARCCE.2015.43147

[34] Papageorgiou EI, Papandrianos NI, Apostolopoulos DJ, Vassilakos PJ. Fuzzy cognitive map based decision support system for thyroid diagnosis management. In: 2008 IEEE international conference on fuzzy systems (IEEE world congress on computational intelligence). IEEE; 2008. p. 1204–1211.

[35] Chatton A, Le Borgne F, Leyrat C, Gillaizeau F, Rousseau C, Barbin L, et al. G-computation, propensity score-based methods, and targeted maximum likelihood estimator for causal inference with different covariates sets: a comparative simulation study. Scientific reports. 2020;10(1):9219. doi: 10.1038/s41598-020-65917-x 32514028 PMC7280276

[36] De Winkel KN, Katliar M, Diers D, Bülthoff HH. Causal inference in the perception of verticality. Scientific reports. 2018;8(1):5483. doi: 10.1038/s41598-018-23838-w 29615728 PMC5882842

[37] Shen X, Ma S, Vemuri P, Simon G. Challenges and opportunities with causal discovery algorithms: application to Alzheimer’s pathophysiology. Scientific reports. 2020;10(1):2975. doi: 10.1038/s41598-020-59669-x 32076020 PMC7031278

[38] Imbens GW, Rubin DB. Rubin causal model. In: Microeconometrics. Springer; 2010. p. 229–241.

[39] Rubin DB. Causal inference using potential outcomes: Design, modeling, decisions. Journal of the American Statistical Association. 2005;100(469):322–331. doi: 10.1198/016214504000001880

[40] VanderWeele TJ. Concerning the consistency assumption in causal inference. Epidemiology. 2009;20(6):880–883. doi: 10.1097/EDE.0b013e3181bd5638 19829187

[41] Yao L, Chu Z, Li S, Li Y, Gao J, Zhang A. A Survey on Causal Inference. ACM Trans Knowl Discov Data. 2021;15(5). doi: 10.1145/3444944

[42] Athey S, Imbens G. Recursive partitioning for heterogeneous causal effects. Proceedings of the National Academy of Sciences. 2016;113(27):7353–7360. doi: 10.1073/pnas.1510489113 27382149 PMC4941430

[43] Tian L, Alizadeh AA, Gentles AJ, Tibshirani R. A simple method for estimating interactions between a treatment and a large number of covariates. Journal of the American Statistical Association. 2014;109(508):1517–1532. doi: 10.1080/01621459.2014.951443 25729117 PMC4338439

[44] Green DP, Kern HL. Modeling heterogeneous treatment effects in survey experiments with Bayesian additive regression trees. Public opinion quarterly. 2012;76(3):491–511. doi: 10.1093/poq/nfs036

[45] Imai K, Ratkovic M. Estimating treatment effect heterogeneity in randomized program evaluation. The Annals of Applied Statistics. 2013;7(1):443–470. doi: 10.1214/12-AOAS593

[46] Berk R, Olson M, Buja A, Ouss A. Using recursive partitioning to find and estimate heterogenous treatment effects in randomized clinical trials. Journal of experimental criminology. 2021;17(3):519–538. doi: 10.1007/s11292-019-09410-0

[47] O’Neill E, Weeks M. Causal Tree Estimation of Heterogeneous Household Response to Time-Of-Use Electricity Pricing Schemes. Faculty of Economics, University of Cambridge; 2018. 1865.

[48] Athey S, Wager S. Estimating treatment effects with causal forests: An application. Observational Studies. 2019;5(2):37–51. doi: 10.1353/obs.2019.0001

[49] Davis JMV, Heller SB. Using Causal Forests to Predict Treatment Heterogeneity: An Application to Summer Jobs. American Economic Review. 2017;107(5):546–50. doi: 10.1257/aer.p20171000

[50] Elek P, Bíró A. Regional differences in diabetes across Europe–regression and causal forest analyses. Economics & Human Biology. 2021;40:100948. doi: 10.1016/j.ehb.2020.100948 33276258

[51] Kreif N, DiazOrdaz K, Moreno-Serra R, Mirelman A, Hidayat T, Suhrcke M. Estimating heterogeneous policy impacts using causal machine learning: a case study of health insurance reform in Indonesia. Health Services and Outcomes Research Methodology. 2021; p. 1–36.33551670

[52] Chen H, Xing J, Yang X, Zhan K. Heterogeneous Effects of Health Insurance on Rural Children’s Health in China: A Causal Machine Learning Approach. International Journal of Environmental Research and Public Health. 2021;18(18):9616. doi: 10.3390/ijerph18189616 34574541 PMC8466805

[53] Ling Z, Yu K, Zhang Y, Liu L, Li J. Causal learner: A toolbox for causal structure and markov blanket learning. Pattern Recognition Letters. 2022;163:92–95. doi: 10.1016/j.patrec.2022.09.021

[54] Ling Z, Li Y, Zhang Y, Yu K, Zhou P, Li B, et al. A light causal feature selection approach to high-dimensional data. IEEE Transactions on Knowledge and Data Engineering. 2022;35(8):7639–7650.

[55] Shortreed SM, Ertefaie A. Outcome-adaptive lasso: variable selection for causal inference. Biometrics. 2017;73(4):1111–1122. doi: 10.1111/biom.12679 28273693 PMC5591052

[56] Zhao Q, Small DS, Ertefaie A. Selective inference for effect modification via the lasso. Journal of the Royal Statistical Society Series B: Statistical Methodology. 2022;84(2):382–413. doi: 10.1111/rssb.12483 36147733 PMC9491375

[57] Li J, Ma S, Le T, Liu L, Liu J. Causal decision trees. IEEE Transactions on Knowledge and Data Engineering. 2016;29(2):257–271. doi: 10.1109/TKDE.2016.2619350

[58] Austin PC, Stuart EA. Moving towards best practice when using inverse probability of treatment weighting (IPTW) using the propensity score to estimate causal treatment effects in observational studies. Statistics in medicine. 2015;34(28):3661–3679. doi: 10.1002/sim.6607 26238958 PMC4626409

[59] Rubin DB. Estimating causal effects from large data sets using propensity scores. Annals of internal medicine. 1997;127(8_Part_2):757–763. doi: 10.7326/0003-4819-127-8_Part_2-199710151-00064 9382394

[60] Xu Z, Cheng D, Li J, Liu J, Liu L, Yu K. Causal Inference with Conditional Front-Door Adjustment and Identifiable Variational Autoencoder. In: ICLR; 2024.

[61] Fulcher IR, Shpitser I, Marealle S, Tchetgen Tchetgen EJ. Robust inference on population indirect causal effects: the generalized front door criterion. Journal of the Royal Statistical Society Series B: Statistical Methodology. 2020;82(1):199–214. doi: 10.1111/rssb.12345 33531864 PMC7845925

[62] Cheng D, Xu Z, Li J, Liu L, Liu J, Le TD. Causal inference with conditional instruments using deep generative models. In: AAAI. vol. 37; 2023. p. 7122–7130.

[63] Baiocchi M, Cheng J, Small DS. Instrumental variable methods for causal inference. Statistics in medicine. 2014;33(13):2297–2340. doi: 10.1002/sim.6128 24599889 PMC4201653

[64] Hill JL. Bayesian nonparametric modeling for causal inference. Journal of Computational and Graphical Statistics. 2011;20(1):217–240. doi: 10.1198/jcgs.2010.08162

[65] Dorie V, Perrett G, Hill JL, Goodrich B. Stan and BART for causal inference: Estimating heterogeneous treatment effects using the power of Stan and the flexibility of machine learning. Entropy. 2022;24(12):1782. doi: 10.3390/e24121782 36554187 PMC9778579

[66] Romano JP, Wolf M. Stepwise multiple testing as formalized data snooping. Econometrica. 2005;73(4):1237–1282. doi: 10.1111/j.1468-0262.2005.00615.x

[67] Cameron AC, Miller DL. A practitioner’s guide to cluster-robust inference. Journal of human resources. 2015;50(2):317–372. doi: 10.3368/jhr.50.2.317

