# Supplementary material for: Utilising causal inference methods to estimate effects and strategise interventions in observational health data
Source: PLoS One. 2024 Dec 30;19(12):e0314761. doi: 10.1371/journal.pone.0314761 (PMC11684594; doi:10.1371/journal.pone.0314761)
Supplement: S1 Appendix — (PDF) [file pone.0314761.s001.pdf]

Appendix for "Utilising Causal Inference Methods to Estimate Effects and Strategise Interventions in Observational Health Data"

This Appendix section includes a discussion on how our method accounts for confounding variables, supplementary experiment results, covering tests on the overlap assumption, further heterogeneity analysis in treatment effect, pre-specified subgroup evaluation in HTE, covariate analysis, cohort ranking, and optimal parametric strategies.

1 Controlling for Confounders in Our Method

Our method addresses confounding factors X by utilizing the propensity score $e(X) = P(W = 1 \mid X)$, where W represents the treatment. Without applying a proper adjustment method, a naive estimation of the average potential outcome across the treated group, μ_1, would be expressed as:

$$\begin{aligned}
\mu_1 &= \mathbb{E}[Y \mid W = 1] && (1)\\
&= \sum_{y} y \times p(y \mid W = 1) && (2)\\
&= \sum_{x,y} y \times p(y \mid W = 1, X = x)\, p(x \mid W = 1) && (3)
\end{aligned}$$

As shown in Eq. 3, the bias arising from $p(x \mid W = 1)$, due to the relationship between confounders and treatment, can distort the estimated average potential outcome, particularly when the confounding values differ significantly between the two groups. By incorporating the propensity score, we can adjust the estimated outcome as follows:

$$\begin{aligned}
\tilde{\mu_1} &= \frac{\mathbb{E}[Y \mid W = 1]}{e(X)} && (4)\\
&= \frac{\sum_{x,y} y \times p(y \mid W = 1, X = x)\, p(x \mid W = 1)}{P(W = 1 \mid X)} && (5)\\
&= \frac{1}{p(W = 1)} \sum_{x,y} y \times p(y \mid W = 1, X = x)\, p(x) && (6)
\end{aligned}$$

In Eq. 6, the adjusted average outcome, $\tilde{\mu_1}$, is free from bias caused by the confounder X and treatment W since $p(x \mid W = 1)$ in Eq. 3 is replaced by $p(x)$. Similarly, in the case of binary treatment, the control group can be adjusted using $1 - e(X)$, as shown in Eq. 4 in our main text. As the adjusted formula incorporates $p(x)$, reflecting the entire confounder distribution, the propensity score balances the distribution of confounders X between the treatment and control groups when computing the effects. This guarantees that any differences in outcome Y are attributed to the treatment rather than the confounders, ensuring a reliable and unbiased estimation.

Table 1. Assessing whether heterogeneity exists and is captured in the causal forest model.

	Estimate	Standard Error	t-value	p-value
Mean Forest Prediction	0.996	0.081	12.322	2.2×10^{-16}
Differential Forest Prediction	0.932	0.130	7.152	4.5×10^{-13}

2 Testing the Overlap Assumption

Before training the causal tree and forest models, we need to test the overlap assumption. This can be done by training a propensity score model $\hat{p}(x) = P(W = 1 \mid X = x)$. Fig. 1 shows the distribution of $\hat{p}(x)$. As required by this assumption, the tails of the distribution do not reach the extremes of 0 and 1, thereby allowing the overlap assumption to be satisfied.

Fig 1. Histogram of the Propensity Score $\hat{p}(x)$. The tails of the distribution do not extend too close to 0 or 1, permitting the overlap condition to hold.

3 Heterogeneity Detection in Treatment Effect

A crucial step is to verify that the learnt casual forest has captured the heterogeneity, if any, can be found across individuals. To do this, following [1], we employ linear predictor analysis with out-of-bag predictions to perform a hypothesis test that compares the predictions generated by the learnt causal forest with the actual responses observed in the data.

For this test, the coefficient of the Mean Forest Prediction assesses whether the forest's average prediction is correct (if this term is close to 1). If the coefficient of the Differential Forest Prediction term is close to 1, the forest has adequately captured any underlying heterogeneity. The p-value of this term, if significant, indicates presence of heterogeneity. Shown in Table 1 are the results of this test. For our data, our causal forest model has been able to both correctly predict the average treatment effect as well as identify and capture the presence of heterogeneity.

4 HTE: Pre-specified Sub-groups

Here, first we estimate the treatment effect difference between two groups for each grouping strategy using the binary covariate "sex". According to the result illustrated in Table 2, males and females respond differently to the treatment in the most visible way, where the BMI reduction for females is estimated to be greater than that of males by 1.031 units, with a low p-value of 0.

Additionally, for multiple hypothesis testing, we examine the treatment effect differences between four education levels in Table 3. The results demonstrate that people with lowest education qualifications react more positively to treatment, with the BMI reduction being 0.873 units better than the figure for those who have bachelor's or post-graduate degrees at a high significance, evidenced by a low p-values of 0.05. Meanwhile, the difference between people who did not complete high school and those who posses certificates or diplomas is least considerable, with a high p-values of 0.676.

Table 2. Heterogeneity Treatment Effect testing for subgroups based on binary covariates.

	Difference	Standard Error	t-value	p-value
Sex (Male v.s. Female)	1.031	0.241	4.286	0

Table 3. Heterogeneity Treatment Effect testing for subgroups based on education level. Group 1: post graduate degree, Group 2: bachelor degree, Group 3: high school, Group 4: secondary school and below. The adjusted p-values are obtained using the Romano-Wolf correction.

	Difference	Standard Error	Original p-value	Adjusted p-value
Group 2 v.s. Group 1	0.527	0.459	0.251	0.393
Group 3 v.s. Group 1	0.164	0.383	0.669	0.676
Group 4 v.s. Group 1	0.873	0.383	0.023	0.05

5 Covariates Analysis

5.0.1 Variable Importance

Through causal forests, a better understanding of a variables importance in determining the impact on treatment effect can be observed. Table 4 shows the variables importances in terms of the percentage of splits in branches across the trees of the forest were made by a particular covariate. In the case of this work, Age was used to split branches 49% of the time and is considered a key variable influencing the treatment effect.

It is important to remember that some variables may be correlated with others and its true importance may not be reflected appropriately. Fig. 2 depicts the correlation between variables. It can be seen that there exists a notable correlation between Age Group and Working Time.

Table 4. Variable Importance.

	Importance
Age Group	0.49
Sex	0.09
Education Level	0.08
Sugar Consumption	0.07
Socio-economic Decile	0.07
Income Decile	0.06
Working Time	0.06
Fiber Consumption	0.05
Remoteness	0.03

Fig 2. Correlation between variables.

Fig 3. Individuals ranked into five equally sized cohorts based on treatment effect. Q1 is largest response and Q5 is smallest response.

6 Cohort Ranking

We now use causal forests to divide our participants into cohorts based on their predicted treatment effect. In this example, we divide into five cohorts. Fig. 3 shows the average treatment effect and confidence for each of these cohorts, with Q1 having the strongest treatment effect. A test of whether the causal forest has been able to accurately model heterogeneity can be checked by observing whether the plot is monotonic as the cohort increases. From this, we can then examine the types of individuals that fall into each cohort by examining the average value of each covariate within the cohorts, as shown in Fig. 4.

6.0.1 Partial Effects

A further benefit of causal forests is that partial effect analysis can be conducted. By holding all but either one or two covariates constant, one can better understand the change in treatment effect for changes in the non-constant covariates.

Keeping all other covariates at their median values, Fig. 5 depicts the impact that differing Age groups have on the average treatment effect, while Fig. 6 examines the influence of Age and Sex on the average treatment effect estimate.

7 Optimal Parametric Strategies

The policy trees of depths one, two, and three are respectively depicted in Fig. 7, Fig. 8, and Fig. 9.

References

1. Cameron AC, Miller DL. A practitioner's guide to cluster-robust inference. Journal of human resources. 2015;50(2):317–372. 3

Fig 4. Average covariate values within five cohorts based on CATE estimated ranking. Q1 is 20% of data with the most significant treatment effect, while Q5 is the 20% of data with the worst improvement in treatment effect.

Fig 5. 95% Confidence interval estimates of average treatment effect over the range of age groups. All other covariates at kept at their median value.

Fig 6. Predicted average treatment effect over the range of Age groups for each Sex level (Male = 1 and Female = 0). Standard error provided in brackets. All other covariates at kept at their median value.

Fig 7. Tree Depth = 1: Optimal Parametric Intervention Targeting Strategy. Left arrow of each split is True, whilst right indicates False. Decision conditions at this depth is if individuals are in Age Group less than or equal to 5 (these are individuals aged 19 or less).

Fig 8. Tree Depth = 2: Optimal Parametric Intervention Targeting Strategy. Left arrow of each split is True, whilst right indicates False. Decision conditions at this depth include whether individuals responded to the Fruit and Vegetable consumption guideline, are in the lowest Socio-economic decile (Socio-economic disadvantage index), and if in Age Group less than or equal to 5 (individuals aged 19 or less).

Fig 9. Tree Depth = 3: Optimal Parametric Intervention Targeting Strategy. Left arrow of each split is True, whilst right indicates False. Decision conditions at this depth include whether Sugar Consumption (Weekly cups of sugar sweetened drinks) is less than equal to 0 or 2, Fiber Consumption (Fruit and Vegetable consumption guideline) was responded to, if in Age Group less than or equal to 5 (aged 19 or less) or 8 (aged 34 or less), and Working Time (Hours usually worked per week) is less than or equal to 24 hours.
