# Supplementary figures and images for: Utilising causal inference methods to estimate effects and strategise interventions in observational health data
Source: PLoS One. 2024 Dec 30;19(12):e0314761. doi: 10.1371/journal.pone.0314761 (PMC11684594; doi:10.1371/journal.pone.0314761)

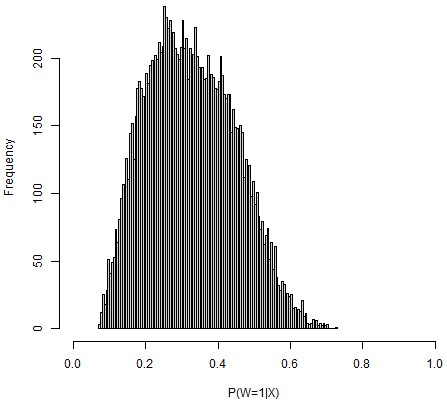

Supplement: S1 Fig — This is used to assess the overlap assumption in our method. (JPG) [file pone.0314761.s002.jpg]

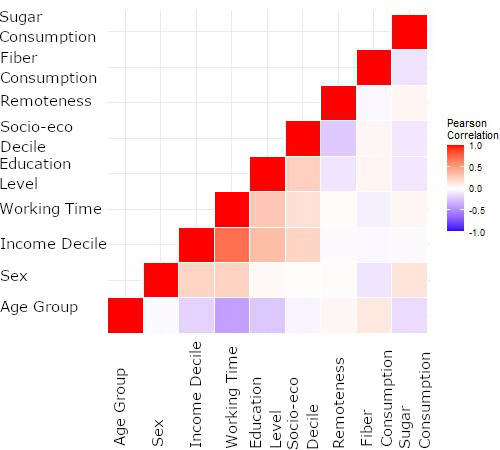

Supplement: S2 Fig — (JPG) [file pone.0314761.s003.jpg]

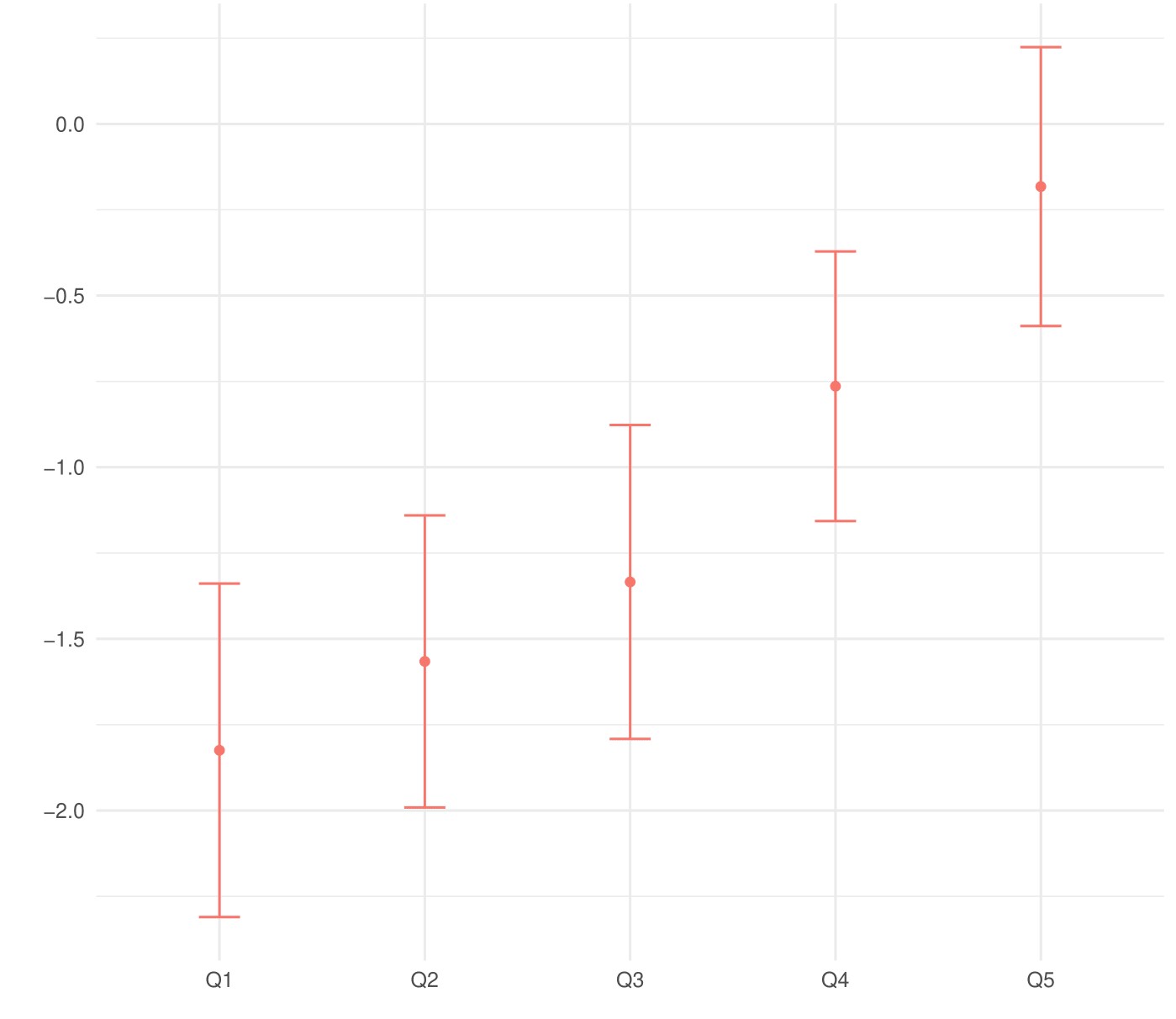

Supplement: S3 Fig — Q1 is largest response and Q5 is smallest response. (JPG) [file pone.0314761.s004.jpg]

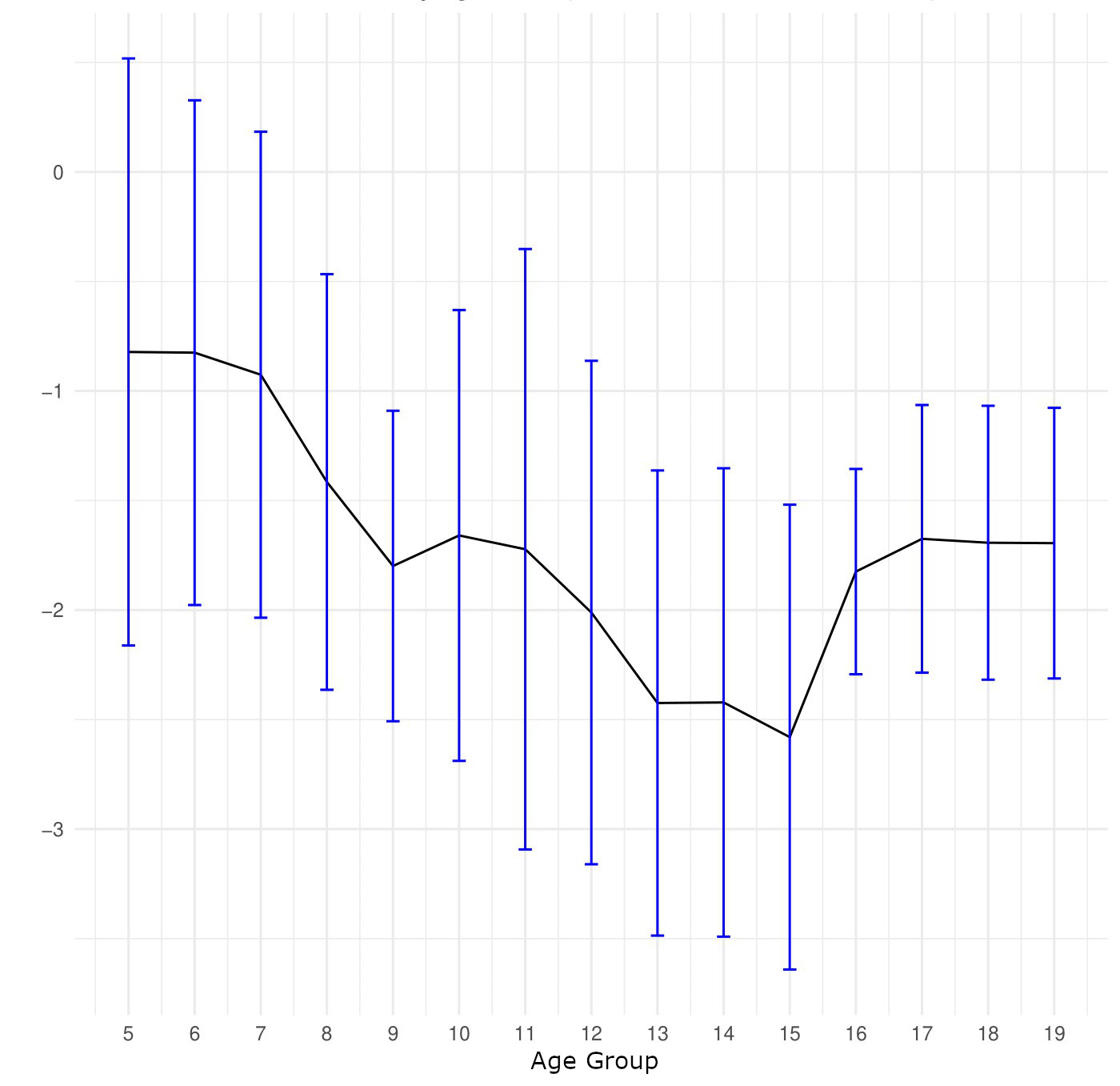

Supplement: S5 Fig — All other covariates at kept at their median value. (JPG) [file pone.0314761.s006.jpg]

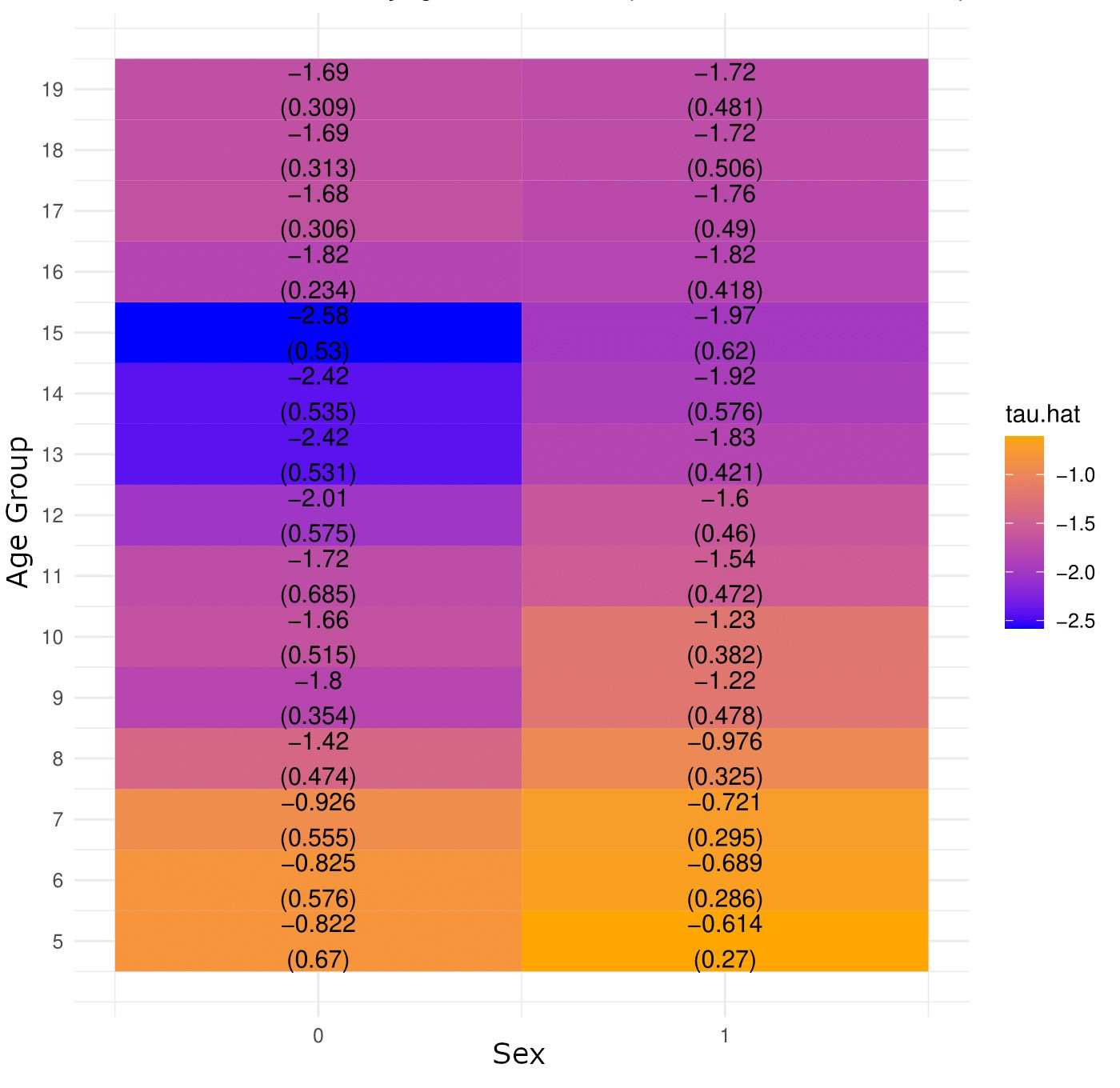

Supplement: S6 Fig — Standard error provided in brackets. All other covariates at kept at their median value. (JPG) [file pone.0314761.s007.jpg]

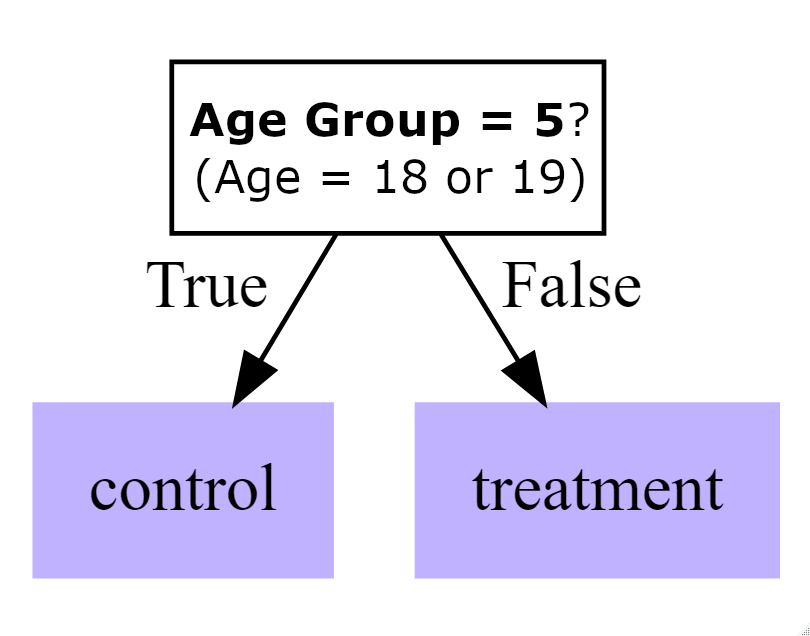

Supplement: S7 Fig — Decision conditions at this depth is if individuals are in Age Group less than or equal to 5 (these are individuals aged 19 or less). (PNG) [file pone.0314761.s008.png]

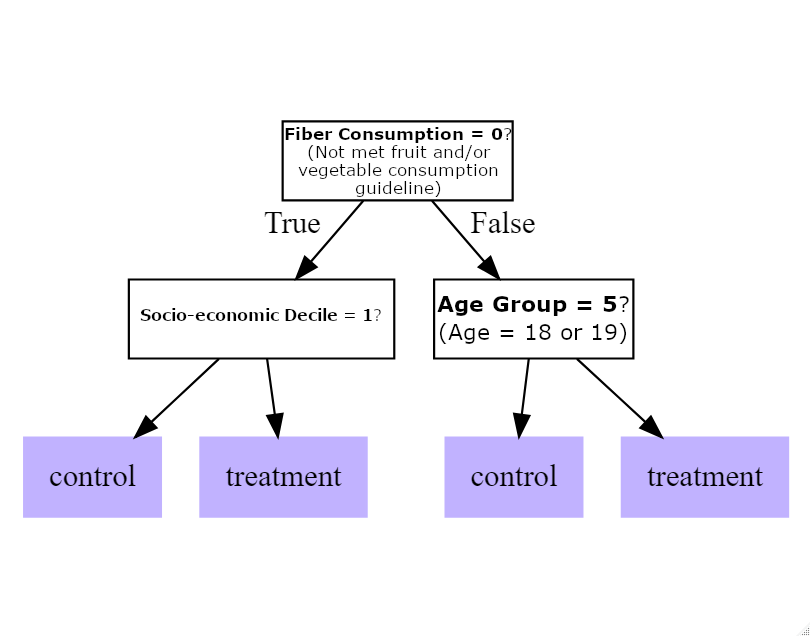

Supplement: S8 Fig — Decision conditions at this depth include whether individuals responded to the Fruit and Vegetable consumption guideline, are in the lowest Socio-economic decile (Socio-economic disadvantage index), and if in Age Group less than or equal to 5 (individuals aged 19 or less). (PNG) [file pone.0314761.s009.png]

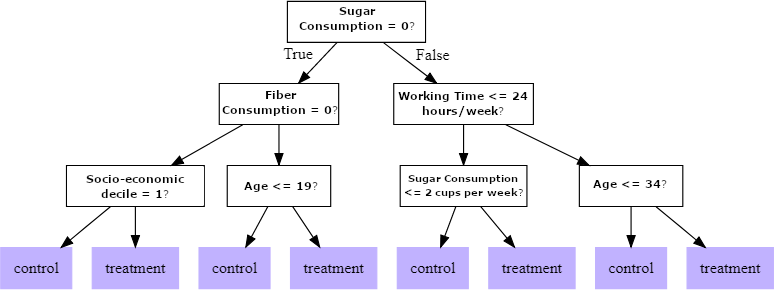

Supplement: S9 Fig — Decision conditions at this depth include whether Sugar Consumption (Weekly cups of sugar sweetened drinks) is less than equal to 0 or 2, Fiber Consumption (Fruit and Vegetable consumption guideline) was responded to, if in Age Group less than or equal to 5 (aged 19 or less) or 8 (aged 34 or less), and Working Time (Hours usually worked per week) is less than or equal to 24 hours. (PNG) [file pone.0314761.s010.png]
