# Supplementary material for: Utilising causal inference methods to estimate effects and strategise interventions in observational health data
Source: PLoS One. 2024 Dec 30;19(12):e0314761. doi: 10.1371/journal.pone.0314761 (PMC11684594; doi:10.1371/journal.pone.0314761)
Supplement: S4 Fig — Q1 is 20% of data with the most significant treatment effect, while Q5 is the 20% of data with the worst improvement in treatment effect. (PDF) [file pone.0314761.s005.pdf]

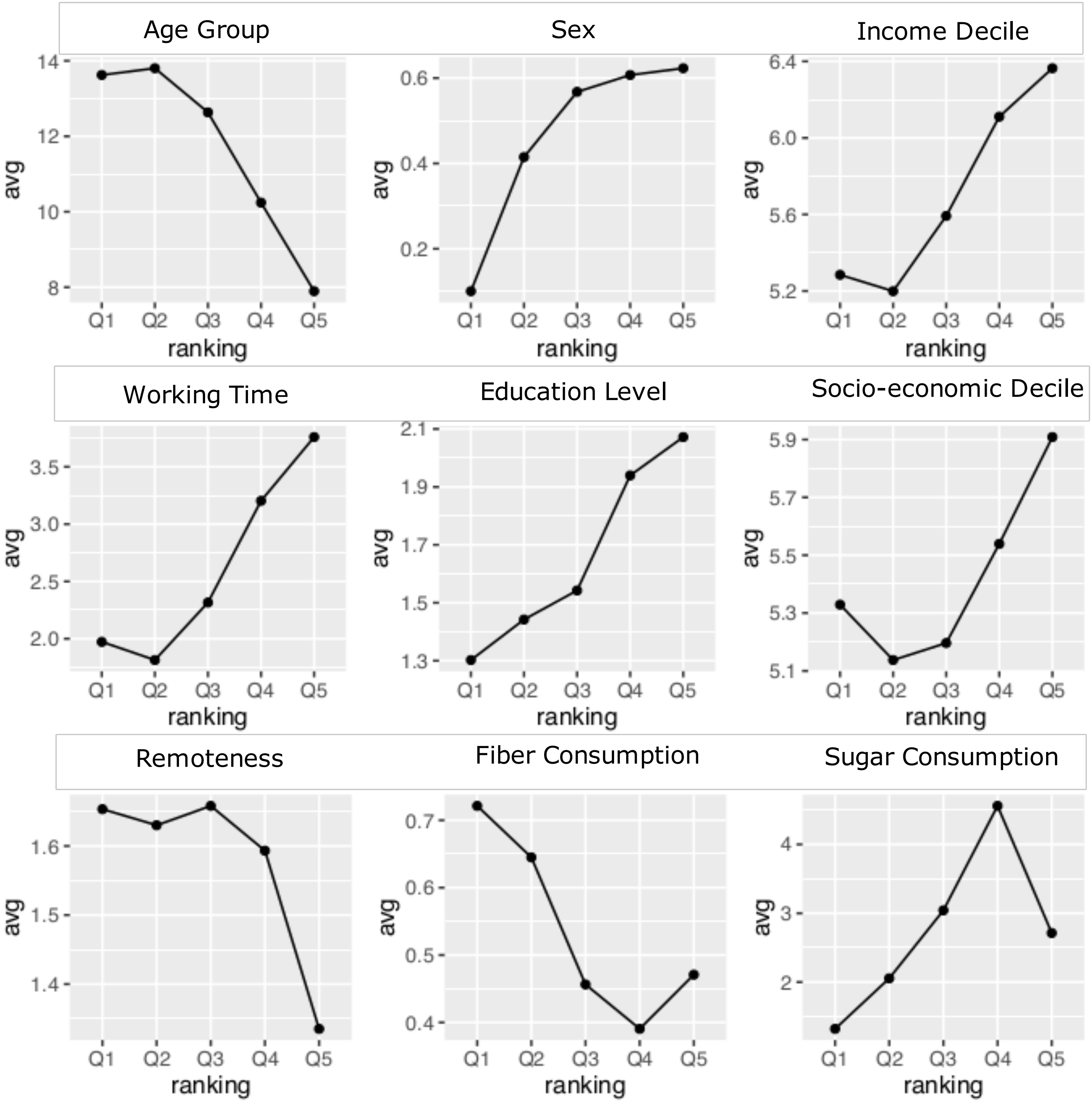

Age Group
Sex
Income Decile
Working Time
Education Level
Socio-economic Decile
Remoteness
Fiber Consumption
Sugar Consumption
avg
ranking
Q1
Q2
Q3
Q4
Q5
